# The Presence of the Y-Chromosome, Not the Absence of the Second X-Chromosome, Alters the mRNA Levels Stored in the Fully Grown XY Mouse Oocyte

**DOI:** 10.1371/journal.pone.0040481

**Published:** 2012-07-06

**Authors:** Baozeng Xu, Yayoi Obata, Feng Cao, Teruko Taketo

**Affiliations:** 1 Departments of Surgery and Biology, McGill University, Montreal, Quebec, Canada; 2 Department of Bioscience, Tokyo University of Agriculture, Tokyo, Tokyo, Japan; Baylor College of Medicine, United States of America

## Abstract

The oocytes of B6.Y^TIR^ sex-reversed female mouse mature in culture but fail to develop after fertilization because of their cytoplasmic defects. To identify the defective components, we compared the gene expression profiles between the fully-grown oocytes of B6.Y^TIR^ (XY) females and those of their XX littermates by cDNA microarray. 173 genes were found to be higher and 485 genes were lower in XY oocytes than in XX oocytes by at least 2-fold. We compared the transcript levels of selected genes by RT-PCR in XY and XX oocytes, as well as in XO oocytes missing paternal X-chromosomes. All genes tested showed comparable transcript levels between XX and XO oocytes, indicating that mRNA accumulation is well adjusted in XO oocytes. By contrast, in addition to Y-encoded genes, many genes showed significantly different transcript levels in XY oocytes. We speculate that the presence of the Y-chromosome, rather than the absence of the second X-chromosome, caused dramatic changes in the gene expression profile in the XY fully-grown oocyte.

## Introduction

The cytoplasm of the oocyte (ooplasm) exerts pivotal and long-lasting effects on not only the meiotic cell cycle progression but also fertilization and preimplantation embryonic development [1], [2]. In the ovary, most of the oocytes are arrested at the end of meiotic prophase until they are recruited into the growth phase accompanied by follicular development. The oocytes are active in transcription during their growth but cease transcription entirely by the end of growth phase [3], [4], [5], [6], and subsequent events are carried out by translation of the stored mRNAs and their post-translational modifications [7], [8], [9]. One of the most critical events in the oocyte is the segregation of homologous chromosomes at the first meiotic division and that of sister chromatids at the second meiotic division. A failure in the proper chromosome/chromatids segregation results in aneuploidy in embryos, the major cause of infertility in women over 35 years old [10], [11]. It is conceivable that some ooplasmic components are required for the proper assembly of meiotic spindles and consequent chromosome segregations. Several molecules have been identified for such roles based on their knock-out or knock-down experiments. However, their phenotypes are often severe and unlikely to occur in the oocytes under physiological conditions such as in older women.

The B6.Y^TIR^ female mouse provides an excellent animal model for investigating the influence of ooplasmic components on the second meiotic spindle assembly and subsequent cell cycle progression under physiological conditions. Sex-reversal occurs when the Y^TIR^-chromosome of a local *Mus. Musculus domesticus* variant in Tirano, Italy (TIR) is placed on the C57BL/6J (B6) genetic background [12], [13]. We have previously shown that the XY female mouse is anatomically normal at young ages, and its oocytes reach the second meiotic metaphase in culture [14]. However, these oocytes contain aberrant second meiotic spindles, which fail at chromosome segregation after fertilization. Furthermore, these meiotic errors can be prevented by transferring the XY nucleus into an enucleated XX oocyte, thereby enabling the production of healthy offspring from the XY oocyte nucleus [15]. The offspring carry various combinations of sex chromosomes such as XO, XXY, and XYY, reflecting the lack of pairing between X- and Y-chromosomes during the meiotic prophase [16], [17]. Thus, sex chromosome aneuploidy per se does not block oocyte maturation or embryonic development while inadequate sex chromosomes appear to render the ooplasm defective.

The XY female also provides a great opportunity to study the role of sex chromosomes in gametogenesis. It is well known that the behavior and transcriptional activity of sex chromosomes in the germ-line are very different between the two sexes. In the female germ-line, the second X-chromosome is reactivated prior to the onset of meiosis and remains active throughout oogenesis [18]. In the male germ-line, on the other hand, both the X- and Y-chromosomes are placed in a compartment, named XY or sex body, and transcriptionally repressed at the pachytene stage of meiotic prophase, named meiotic sex chromosome inactivation (MSCI) [19]. It is, therefore, informative to examine the behavior of sex-chromosomes in the XY oocyte. Our previous studies have shown that the X- and Y-chromosomes do not pair and the majority of single X-chromosomes are silent, as evident by the coating with γH2AX, at the pachytene stage of meiotic prophase [20]. However, the single Y-chromosome is rarely coated with γH2AX and at least one Y-encoded gene, *Ube1y1*, is transcribed in the XY fetal ovary. The transcriptional activity of sex chromosomes in XY oocytes during the resting stage or growth phase has not yet been examined.

In the present study, we compared gene expression profiles between XX and XY oocytes at the GV-stage collected from antral follicles by cDNA microarray. We evaluated the transcript levels of selected genes by semi-quantitative RT-PCR (sqRT-PCR) in XX and XY oocytes, as well as XO oocytes, in which the paternal X-chromosome is absent like XY oocytes. Such XO female mice can be produced from the XY male mouse carrying a *Paf* mutation on its X-chromosome, which result in a high frequency of X-Y non-disjunction [21], [22]. Our results suggest that the expression of Y-encoded genes, rather than the haplodefficiency of X-encoded genes, makes the XY oocyte defective in its cytoplasm.

## Results and Discussion

### Differentially expressed genes between XX and XY oocytes identified by microarray

In order to identify the cytoplasmic components responsible for the developmental incompetence of XY oocytes, we compared the gene expression profiles in the fully-grown oocytes from XY females with those from XX littermates by microarray analysis. The Affymetrix Mouse Expression 430v2.0 GeneChip contains 45,037 probe sets that represent 14,484 full-length genes, 9450 non-ESTs and 21,103 ESTs, according to the manufacturer. The results showed that 4 Y-encoded, 8 X-encoded, and 161 autosomal genes had significantly higher transcript levels, whereas 77 X-encoded and 408 autosomal genes had lower transcript levels in XY oocytes than in XX oocytes by at least 2-fold (P<0.05 by students t-test) (Tables S1 and S2). Of these, 65 genes appeared with two or more probe sets which were available in the microarray, suggesting that results were highly reproducible. The differentially expressed genes include those involved in apoptosis, mitochondrial functions, posttranslational modifications, cellular gap junctions, carbohydrate metabolism, and ubiquitination.

### mRNAs of most Y-encoded genes were detectable in XY oocytes

**Figure 1:**
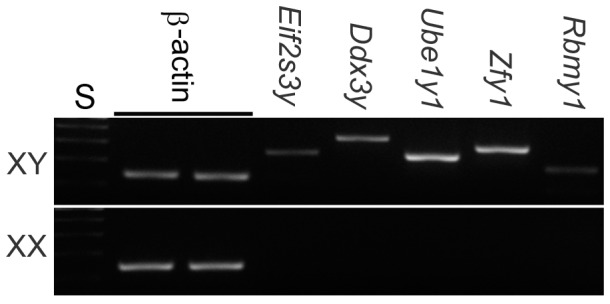
RT-PCR detection of Y-encoded gene transcripts in XY and XX oocytes. Agarose gel electrophoresis stained with ethidium bromide. High transcript levels of *Eif2s3y*, *Ddx3y*, and *Ube1y1* and lower levels of *Rbmy1* were visible in XY oocytes but not in XX oocytes. The transcript levels of *Zfy1* was similar to those of *Rbmy1* after the same amplification cycles, but increased, as shown here, by adding 4 more cycles. For each RT-PCR experiment, β-actin was included as an internal control. S, 100 bp ladders.

Microarray data showed that the transcript levels of Y-encoded genes, *Ddx3y*, *Eif2s3y*, *Ube1y1*, and *Zfy2* were significantly higher in XY oocytes than in XX oocytes by at least 2-fold. We examined the transcript levels of these genes by linear-range sqRT-PCR by using random hexamers for cDNA synthesis. We also examined Y-encoded genes *Rbmy1*, *Sry*, *Ssty1*, *Usp9y*, *Uty*, and *Zfy1*, which were not included as being differentially expressed in the microarray data. Examples of agarose gel electrophoresis are shown in Figure 1. The transcript levels of *Eif2s3y*, *Ddx3y*, *Ube1y1*, and *Uty* (not shown) were high, those of *Rbmy1*, *Ssty1*, *Ups9y*, and *Zfy1* (not shown) were near the detection limit, and those of *Zfy2* or *Sry* (not shown) were undetectable in XY oocytes. The transcript levels of *Zfy*1 were similar to those of *Rbmy1* after the same 36 amplification cycles (not shown), but increased after 4 more cycles (Figure 1). On the other hand, none of the examined genes was detectable in XX oocytes. Results were consistent in three repeated experiments. These results suggest that the Y-chromosome is transcriptionally active in XY oocytes during the growth phase. Since *Rbmy1*, *Sry*, and *Zfy2* are located near the centromere, their low or undetectable levels can be attributed to the heterochromatinization of this region.

*Eif2s3y* and *Ddx3y* encode highly conserved eukaryotic translation initiation factor subunit and RNA helicase, respectively, and are known to be essential for spermatogenesis in the mouse [23], [24], [25], [26]. *Ube1y1* encodes a ubiquitin-activating enzyme and may be involved in protein degradation during the metaphase to anaphase transition [27], [28]. *Rbmy1* encodes a RNA-binding protein, repeated over 50 times, and is known to be expressed in a germ-cell-dependent manner in both fetal and adult testes [29]. *Zfy1*and *Zfy2* are transcription factors encoding zinc-finger proteins, highly homologous but differentially regulated in males; *Zfy2* is predominantly expressed in the germ cells of adult testes [30], [31] whereas *Zfy1* is expressed, in addition, in various embryonic tissues [30], [31], [32]. Although the microarray data included *Zfy2*, sqRT-PCR indicated that *Zfy1* was expressed at much higher levels than *Zfy2* in XY oocytes. It is conceivable that the microarray probe set may have hybridized with *Zfy1* in the nearly absence of *Zfy2*. It was recently suggested that both *Zfy1*and *Zfy2* play a role in the elimination of asynapsed spermatocytes [33].

**Figure 2:**
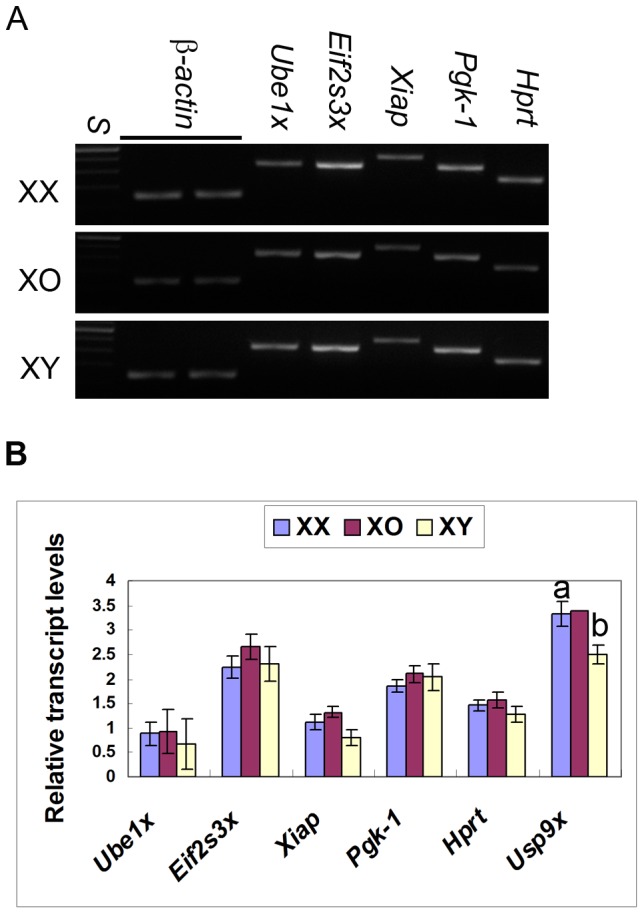
RT-PCR detection of X-encoded gene transcripts in XX, XO and XY oocytes. A. Agarose gel electrophoresis stained with ethidium bromide. S, 100 bp ladders. B. Relative transcript levels. In each set of experiment, the transcript levels were normalized against the mean of two β-actin controls. Each column indicates the mean ± SEM (n = 6 for *Xiap* and n = 3 for others except for *Usp9x* in XO, n = 1). a and b above columns indicate statistical differences at P<0.05 by paired students t-test.

We do not know if any of these Y-encoded gene products exerts direct detrimental effects on the oocyte. However, it is conceivable that the expression of Y-encoded genes, which does not occur in normal oocytes, may cause dynamic changes in the expression of other genes in XY oocytes. For example, the expression of Y-encoded genes may influence the expression of their homologous X-encoded genes. To test this possibility, we compared the transcript levels of *Ube1x (Uba1)*, *Usp9x*, and *Eif2s3x* normalized to those of β-actin in XX and XY oocytes by sqRT-PCR. No difference was found for *Ube1x* or *Eif2s3x* whereas the transcript levels of *Usp9x* was significantly lower in XY oocytes than in XX oocytes (n = 3 each) (Figure 2). Since we found that the transcript levels of *Ube1y* and *Eif2s3y* were high (Figure 1) while those of *Usp9y* were near the detection limit (not shown), there was no direct correlation between the expression of Y-encoded genes and that of their homologous X-encoded genes in the fully-grown oocyte. It is intriguing that the transcript levels of most X-encoded genes in XY oocytes were no less than in XX oocytes despite their carrying single X chromosomes. Therefore, we also examined XO oocytes, but did not find any difference (Figure 2).

### Accumulation of X-encoded gene mRNAs in XX, XO and XY oocytes

**Figure 3:**
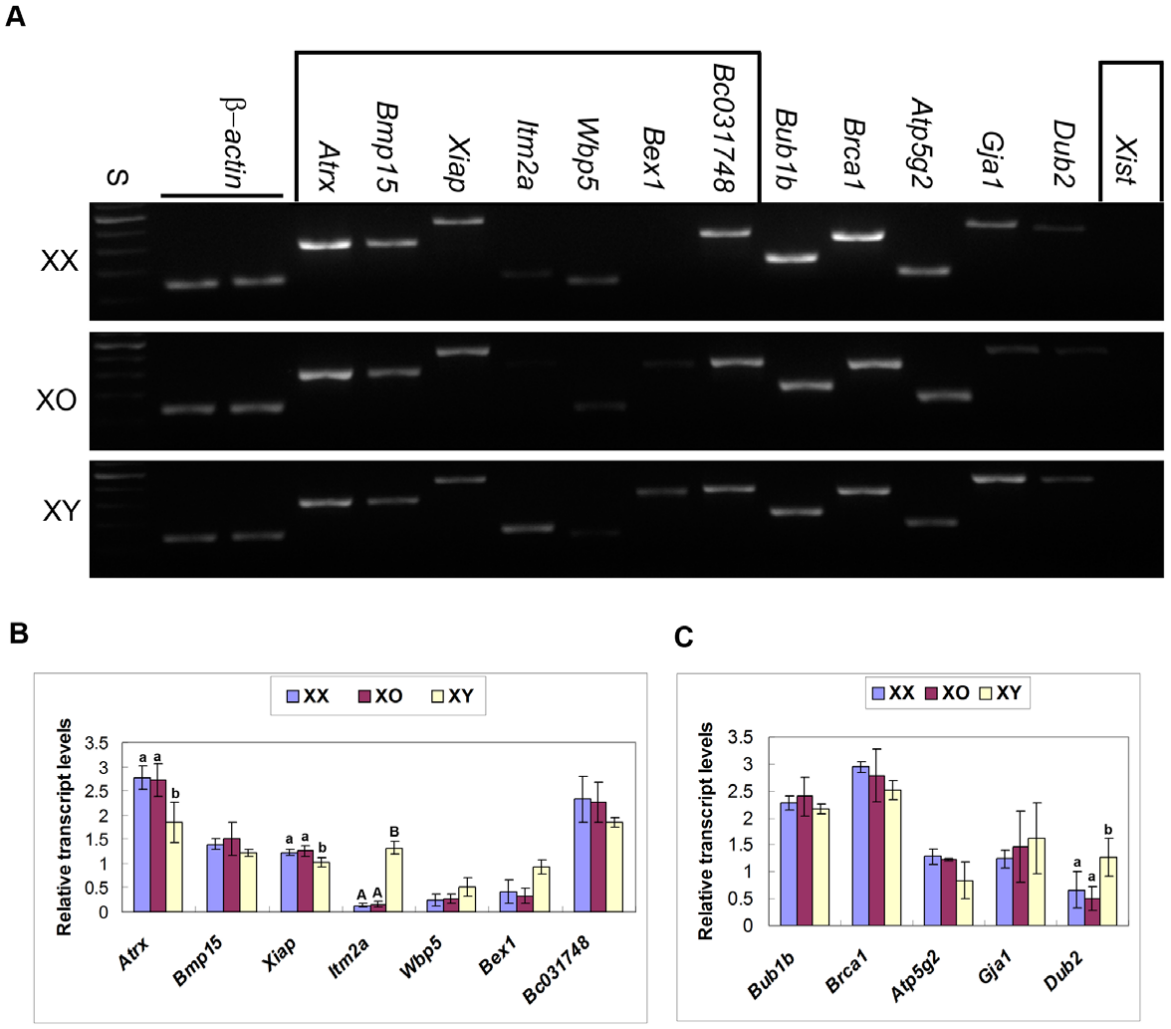
RT-PCR detection of additional X-encoded (in rectangular boxes) and autosomal gene transcripts in XX, XO and XY oocytes. A. Agarose gel electrophoresis stained with ethidium bromide. S, 100 bp ladders. B and C. Relative transcript levels of. X-encoded (A) and autosomal (C) genes. In each set of experiment, the transcript levels were normalized against the mean of two β-actin controls. Each column indicates the mean ± SEM (n = 6 for *Atrx* and *Xiap*, n = 4 for *Atp2g5* and *Dub2*, n = 3 for others). Different low case and capital letters above columns denote statistical differences at P<0.05 and 0.01, respectively, by one-way ANOVA.

The microarray data showed that the transcript levels of seven X-encoded genes were higher in XY oocytes than in XX oocytes by at least 2-fold. To confirm the results of microarray, we examined three selected genes, *Bex1*, *Itm2a*, and *Wbp5*, in XX and XY oocytes by sqRT-PCR. In anticipation of X dosage-dependence, we also included the oocytes from the XO female carrying the maternal X-chromosome in these studies. As shown in Figure 3, the transcript levels of *Itm2a* were significantly higher in XY oocytes than in XX or XO oocytes (n = 3, P<0.01). The levels of *Bex1* and *Wbp5* were also higher but did not reach a significant difference (n = 3). Microarray analysis also showed that the transcript levels of *Atrx*, *Bmp15*, and *Xiap* were lower in XY oocytes than in XX oocytes by at least 2-fold. sqRT-PCR analysis confirmed that the transcript levels of *Atrx* and *Xiap* were significant lower in XY oocytes than in XX or XO oocytes (n = 6, P<0.05). No difference was found for *Bmp15* (n = 4). We added two X-encoded housekeeping genes, *Pgk-1* and *Hprt*, but again found no difference between XX, XO and XY oocytes (Figure 2). We used transcript levels of β-actin for normalizing the results in each pool of oocytes. No difference was found in the β-actin transcript levels in the same number of fully-grown oocytes from XX, XO, or XY females (n = 3) (not shown).

It is known that the second X-chromosome in the XX oocyte is activated prior to the onset of meiosis and remains active throughout oogenesis [18]. This is in agreement with our data that no *Xist* mRNAs were detectable in XX oocytes at the GV-stage (Figure 3). Consequently, we anticipated the expression of X-encoded genes in XO and XY oocytes to be half of those in XX oocytes. Therefore, we were surprised that the transcript levels of most X-encoded genes were comparable in XX, XO, and XY oocytes. The discrepancy between the microarray data and sqRT-PCR results can be attributed to the choice of primers, oligo-d(T) and random hexamers, respectively, for cDNA synthesis; Oligo-d(T) primers are less efficient in the reverse transcription of mRNAs with short poly(A) tails, which are common in the fully grown oocyte. We speculate that we detected the accumulation of mRNAs, not the steady-state transcription, in the fully-grown oocyte [34], [35]. Therefore, our results suggest that the maximum storage of mRNAs is adjusted in XX and XO oocytes. In this light, significantly higher or lower transcript levels of some X-encoded genes in XY oocytes, namely *Itm2a*, *Atrx*, and *Xiap*, are remarkable and may be associated with the cytoplasmic defects of XY oocytes.

*Itm2a* encodes a type II integral membrane protein that is involved in osteo- and chondrogenic differentiation [36]. Its expression or role in germ cells is unknown. Since it contains consensus binding sites for various transcription factors, its transcript levels may have been altered by a change in certain transcription factors in XY oocytes. ATRX, a member of the SNF2 family of helicase/ATPase, is required for the second metaphase (MII) chromosome alignment and meiotic spindle assembly in mouse oocytes [37]. It was also reported that ATRX deficiency results in centromere instability and a high incidence of aneuploidy in oocytes and preimplantation embryos [38]. In the XY oocyte matured in vitro, we have observed an aberrant MII-spindle with chromosome misalignment, leading to a failure in the second meiotic division after fertilization [17]. We speculate that the lower *Atrx* transcript levels may be, at least partially, responsible for the meiotic defects in the XY oocyte. *Xiap* encodes a dominant endogenous inhibitor of apoptosis [39]. The direct role of XIAP in protecting oocytes from apoptosis is unlikely since XY oocytes within follicles rarely undergo apoptosis [40]. However, the majority of follicles undergo atresia under physiological conditions, and high expression of XIAP in follicular cells is associated with the prevention of follicular atresia [41], [42], [43]. Therefore, lower *Xiap* transcript levels in the XY oocyte may reflect the status in its neighbouring cumulus cells.

### Transcript levels of autosomal genes in XX, XO, and XY oocytes

**Figure 4:**
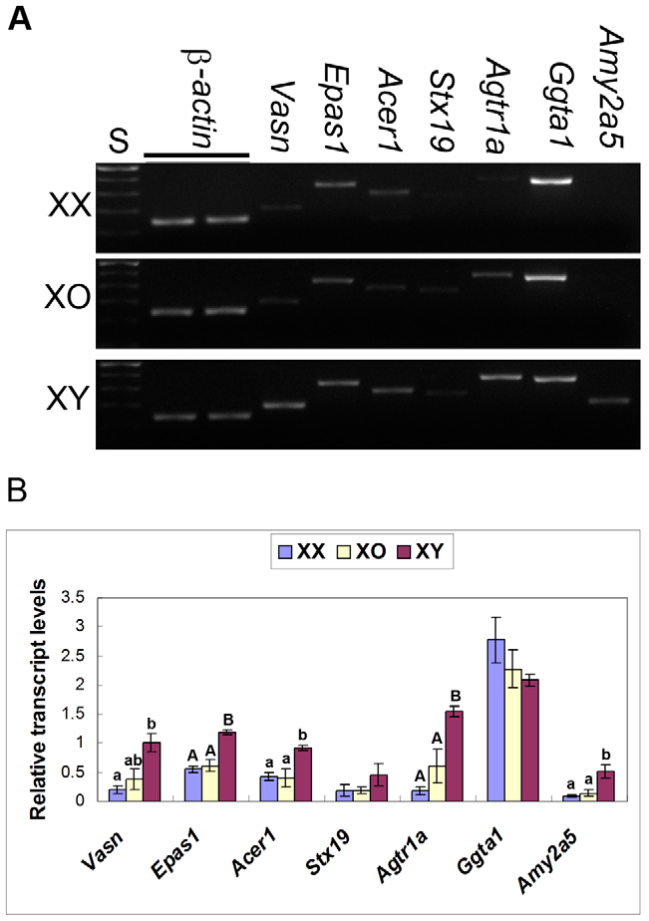
RT-PCR detection of additional autosomal gene transcripts in XX, XO and XY oocytes. A. Agarose gel electrophoresis stained with ethidium bromide. S, 100 bp ladders. B. Relative transcript levels of autosomal genes in XX, XO and XY oocytes. In each set of experiment, the transcript levels were normalized against the mean of two β-actin controls. Each column indicates the mean ± SEM (n = 3). Different low case and capital letters above columns denote statistical differences at P<0.05 and 0.01, respectively, by one-way ANOVA.

The results of microarray analysis showed that the transcript levels of 179 genes spread over all autosomes were higher in XY oocytes than in XX oocytes by at least 2-fold. sqRT-PCR analysis confirmed that, from the list of differentially expressed genes, *Agtr1a*, *Amy2a1/2a5/2b*, and *Vasn* were significantly higher in XY oocytes than in XX or XO oocytes in the range of 10–15 fold (Figure 4). *Stx19* transcript levels were higher but did not reach a statistical significance (n = 3). The microarray data also showed that the transcript levels of *Acer1*, *Dub2*, and *Epas1* were higher in XY oocytes but they were eliminated from the final list because signals of some samples were not within the acceptable range. However, sqRT-PCR confirmed that they were significantly higher in XY oocytes (Figures 3 and 4). We tested more genes, such as *Bub1b*, *Brca1*, *Gja1*, and *Ggta1*, which were not included in the miscroarray data as differentially expressed genes, and sqRT-PCR confirmed that their transcript levels were similar in XY and XX oocytes. Transcript levels of all genes examined were comparable between XX and XO oocytes. These results are consistent with our hypothesis that the storage of mRNAs for most genes was well adjusted in the fully grown oocyte, but those for specific genes were abnormally higher in XY oocytes.

**Figure 5:**
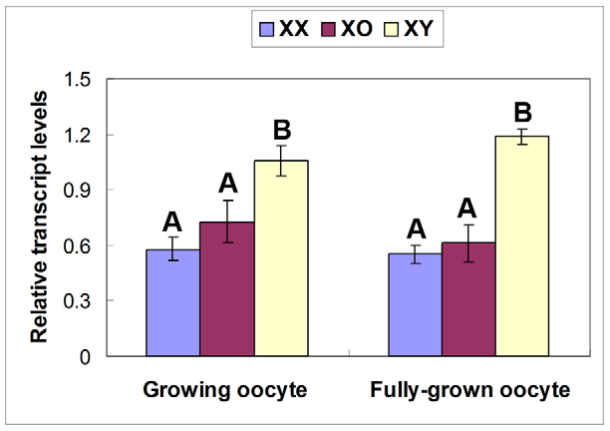
Relative transcript levels of *Epas1* in XX, XO and XY oocytes collected from ovaries at 10 and 30 days after birth. “Growing oocytes” were selected for their diameter between 40 and 50 µm. “Fully grown oocytes” were denuded from oocyte-cumulus complexes of antral follicles. The transcript levels were normalized against the mean of two β-actin controls. Each column indicates the mean ± SEM (n = 3). A and B above columns denote statistical differences at P<0.01 by paired students t-test.

The higher transcript levels of *Amy2a5* and *Epas1* may reflect the poor supply of energy source into the XY oocyte from its neighboring cumulus cells. *Amy2a5* encodes pancreatic α-amylase, which is involved in the metabolism of carbohydrate and fatty acids such as starch, glycogen, and oligosaccharides. α-glucosidase, a resultant metabolite, further degrades disaccharides into simpler sugars [44], which are readily available for ATP production. It is known that *Amy2a5* is upregulated in the absence of glucose in certain cell types [45]. Since oocytes cannot directly use glucose, the surrounding cumulus cells must provide oocytes with glucose metabolites [46]. Therefore, our present results may indicate that either the oocyte-cumulus cell communication is impaired or cumulus cells are malfunctioning in the XY ovary. *Amy2a5* appeared to be upregulated shortly before the end of growth phase since its transcripts were undetectable in the growing oocytes isolated from XY ovaries at 17 days postpartum (dpp) (not shown). *Epas1* encodes a transcription factor, Hypoxia-Inducible Factor 2α (HIF-2α), and plays key roles in the Krebs cycle and fatty acid oxidation [47]. Its upregulation in XY oocytes may reflect a limited energy supply from the cumulus cells. Unlike *Amy2a5*, however, *Epas1* transcript levels were significantly higher in the growing oocytes isolated from XY ovaries as early as 10 dpp (Figure 5). It is conceivable that the altered gene expression profile in XY oocytes may not be cell-autonomous but involve interaction with the surrounding somatic cells.

*Agtr1a*, *Epas1*, and *Vasn* are known to play roles in ovulation [48], [49], [50], [51], [52]. We have previously reported that follicular development is largely normal up to the antral stage but rarely reaches the preovulatory stage in the XY ovary [40], and very few or no oocytes are ovulated by XY females by gonadotropin treatment [53]. The higher transcript levels of the above three genes in XY oocytes may reflect the conditions which are essential for ovulation. *Dub2* encodes a deubiquitinating enzyme involved in proteolysis, which has been implicated in the control of mammalian gametogenesis and fertilization [54], [55], [56]. *Acer1* encodes alkaline ceramidase 1, which catalyzes hydrolysis of ceramides to generate sphingosine (SPH), which in turn is phosphorylated to form sphingosine-1-phosphate (S1P). Ceramide, SPH, and S1P are bioactive lipid second messengers associated with cell proliferation and apoptosis [57], [58], [59], [60], [61]. The higher transcript levels of *Acer1* in XY oocytes may influence the proliferation of cumulus cells. This is consistent with our previous finding that the number of cumulus cells in the antral follicles is smaller in the XY ovary than in the XX ovary by 40% [62].

### Conclusion

The present results show that similar levels of mRNAs were accumulated in XO and XX oocytes by the end of growth phase despite the different dosages of X-encoded genes. By contrast, mRNA levels of certain X-encoded and autosomal genes were significantly different in XY oocytes. We speculate that the accumulation of mRNAs was adjusted despite the absence of the second X-chromosome, but altered by the expression of Y-encoded genes in the fully-grown oocyte. We need further studies to provide evidence for supporting this hypothesis. Nonetheless, the list of genes in the present study provides an opportunity to identify the ooplasmic components which have critical effects on the second meiotic spindle assembly in the oocyte.

## Materials and Methods

### Mouse

All animal experiments were conducted in accordance with the Guide to the Care and Use of Experimental Animal issued by the Canadian Council on Animal Care and with the approval by Animal Research Committee of McGill University. The B6.Y^TIR^ mouse was established by repeating backcrosses to place the Y-chromosome of TIR on the B6 genetic background [13]. B6.Y^TIR^ male mice (N45–50 backcross generations) were crossed with B6 females (Jackson Laboratory, Bar Harbor, ME) to produce XY females and their XX littermates. A *Paf* carrier mutant male on the C3Heb/Fej background was purchased from the Jackson Laboratory and backcrossed to B6 females in our mouse colony. *Paf* carrier males (8–10 backcross generations) were crossed with B6 females to produce XO females carrying maternal X-chromosomes. The day of delivery was defined as 0 dpp.

### Genotyping

Upon weaning of pups at 20–25 dpp, their ear punches were taken and used for determining their genotypes. For the offspring from the cross between B6 females and B6.Y^TIR^ males, ear punches were digested overnight in a lysis buffer and the heat-inactivated lysate was subjected to PCR amplification of the Y-encoded *Zfy* gene as described previously [63]. For the offspring from the cross between B6 females and *Paf* carrier males, total RNA was extracted from ear punches by using TRIzol (Invitrogen, Burlington, ON) and dissolved in RNase-free water. The RNA sample was then subjected to cDNA synthesis and subsequent PCR amplification of the *Xist* transcript, using the conditions and primers previously described [64]. *Xist* is transcribed in XX females but not in XO or XY females.

### Collection of oocytes

XX, XY and XO females at 25–29 dpp were injected intraperitoneally each with 5 IU equine chorionic gonadotropin (Sigma, St Louis, MO), and sacrificed 45–47 hours later. Fully grown GV-stage oocytes surrounded by cumulus cells were isolated by puncturing large antral follicles with a pair of 26-gauge needles, and then denuded of cumulus cells by repeated pipetting through a fine glass needle. Only the oocytes with an intact GV and no apparent sign of degeneration were collected and stored at −80°C. Growing oocytes were collected from XX, XY, and XO females at 10 and 17 dpp. Ovaries were treated with 0.5 mg/ml each of testicular hyaluronidase (type IV), collagenase (B grade) and egg white lysozyme (all from Sigma) in M2 medium as previously reported [65]. Oocytes with 40–60 µm diameter were collected with a fine glass needle, transferred in a group into a microcentrifuge tube, and stored at −80°C.

### Microarray analysis

Total RNA were isolated from 10 oocytes each from 3 XY and 3 XX females using the RNeasy Micro Kit (Qiagen, Valencia, CA) and extracted in 11 µl RNase-free water. The Two-Cycle Eukaryotic Target Labeling Kit (Affymetrix) was used for synthesizing cRNA with oligo(dT) primers starting from 9 µl total RNA in solution. Manufactures' protocols were optimized for small quantities of total RNA as previously reported [66]. Equal amounts (10 µg) of fragmented and biotin-labeled cRNA from each sample were hybridized to Affymetrix Mouse Genome 430 v2.0 GeneChip® arrays for 16 h at 45°C. The GeneChips were then washed and stained with a GeneChip Fluidics Station 460 (Affymetrix) according to the Expression Analysis Technical Manual. An Affymetrix GeneChip Scanner 3000 was used to quantify the signals. The GeneChip Operating Software (GCOS) version 1.3 (Affymetrix) output files were then loaded into GeneSpring v10.1 (Agilent Technologies, Santa Clara, CA) with a normalization shift to the 50th percentile and baseline transformation to the median of all the samples. In the first step of data processing, present (P), marginal (M), or absent (A) calls, based on hybridization intensity with perfect match probes over that with mismatch probes, were used to filter gene lists, and transcripts with a raw signal intensity greater than 100 for at least two samples in either XX or XY group were selected. In the second step, genes with at least 2-fold differences common in three sample sets were selected. In the resultant list, significant differences (P<0.05) were calculated between XX and XY samples by students t-test. When one sample of the group with higher expression was below 100 (A call), it was excluded, while all three samples of the group with lower expression were included in the statistical analyses.

### Semi-quantitative RT-PCR

Total RNA were isolated from 40–80 oocytes in a group using the RNeasy Micro Kit as described above. First-strand cDNA synthesis was carried out by using Moloney Murine Leukemia Virus Reverse Transcriptase (M-MLV, Invitrogen) and random hexamers as described previously [67]. Each cDNA sample was processed for PCR amplification by denaturation at 95 °C for 5 min followed by 36 cycles (unless specified in Table S3) at 95 °C for 25 sec, 60 °C for 25 sec, and 72 °C for 40 sec and final extension at 72 °C for 10 min with a thermocycler (Biometra, Model T1, Göttingen, Germany). The primers used for RT-PCR are given in Table S3. After PCR amplifications, 15 µl of each reaction mixture was applied to 2% agarose gel electrophoresis in TAE buffer and visualized with ethidium bromide fluorescence labeling. The intensity of each band was quantified and the relative intensity against β-actin in the same lane was calculated by using AlphaImager 2000 system (Alpha Innotech Co., San Leandro, CA). Results were analyzed by either one-way ANOVA followed by LSD test or students t-test.

## Supporting Information

Table S1 **Differentially expressed genes at higher levels in XY oocytes than in XX oocytes, identified by cDNA microarray.** This table provides the list of genes which were found to be expressed at higher levels in XY oocytes than in XX oocytes by at least 2-fold (p<0.05, student t-test).
(DOC)

Table S2 **Differentially expressed genes at lower levels in XY oocytes than in XX oocytes, identified by cDNA microarray.** This table provides the list of genes which were found to be expressed at lower levels in XY oocytes than in XX oocytes by at least 2-fold (p<0.05, students t-test).
(DOC)

Table S3 **Primer sequences.** This table provides the list of primer sequences used for RT-PCR.
(DOC)
