# Correction: The Presence of the Y-Chromosome, Not the Absence of the Second X-Chromosome, Alters the mRNA Levels Stored in the Fully Grown XY Mouse Oocyte

**DOI:** 10.1371/journal.pone.0143788

**Published:** 2015-11-23

**Authors:** Baozeng Xu, Yayoi Obata, Feng Cao, Teruko Taketo

The probe ID numbers given in Table S1 and Table S2 are incorrect. The correct probe ID numbers are available from: http://www.ncbi.nlm.nih.gov/geo/query/acc.cgi?acc=GSE45668.
