# Supplementary material for: The Presence of the Y-Chromosome, Not the Absence of the Second X-Chromosome, Alters the mRNA Levels Stored in the Fully Grown XY Mouse Oocyte
Source: PLoS One. 2012 Jul 6;7(7):e40481. doi: 10.1371/journal.pone.0040481 (PMC3391287; doi:10.1371/journal.pone.0040481)
Supplement: Table S1 — Differentially expressed genes at higher levels in XY oocytes than in XX oocytes, identified by cDNA microarray. This table provides the list of genes which were found to be expressed at higher levels in XY oocytes than in XX oocytes by at least 2-fold (p<0.05, student t-test). (DOC) [file pone.0040481.s001.doc]

Table S1. Differentially expressed genes identified by cDNA microarray analysis

**Higher in XY oocytes than in XX oocytes by at least 2-fold**

Gene symbol Gene name Chromosome Probe set ID

Abca1 ATP-binding cassette, sub-family A (ABC1), member 1 chr4 1433865_at

Acaa2 acetyl-Coenzyme A acyltransferase 2 chr18 1433930_at

Acap1 ArfGAP with coiled-coil, ankyrin repeat and PH domains 1 chr11 1433944_at

Acox1 acyl-Coenzyme A oxidase 1, palmitoyl chr11 1433992_at

Acsm2 acyl-CoA synthetase medium-chain family member 2 chr7 1434022_at

Actr1b ARP1 actin-related protein 1 homolog B, centractin beta (yeast) chr1 1434030_at

Afp alpha fetoprotein chr5 1434076_at

1434283_at

Agtr1a angiotensin II receptor, type 1a chr13 1434285_at

Ak7 adenylate kinase 7 chr12 1434294_at

Alx1 ALX homeobox 1 chr10 1434301_at

Amy2a4, 2a5 amylase 2a4, 2a5 chr3 1434322_at

Ankrd24 ankyrin repeat domain 24 chr10 1434394_at

Arih2 ariadne homolog 2 (Drosophila) chr9 1434398_at

Asb13 ankyrin repeat and SOCS box-containing 13 chr13 1434446_at

Atp6v1e1 ATPase, H+ transporting, lysosomal V1 subunit E1 chr6 1434477_at

Atxn7l1 ataxin 7-like 1 chr12 1434478_at

B3galtl beta 1,3-galactosyltransferase-like chr5 1434497_at

B4galt3 UDP-Gal:betaGlcNAc beta 1,4-galactosyltransferase, polypeptide 3 chr1 1434613_at

Bcl2l15 Bcl2-like 15 chr3 1434873_a_at

Bex1 brain expressed gene 1 chrX 1434969_at

Bhlhb9 basic helix-loop-helix domain containing, class B9 chrX 1434978_at

C1d C1D nuclear receptor co-repressor chr11 1434982_at

Calb1 calbindin 1 chr4 1435022_at

Cbr3 carbonyl reductase 3 chr16 1435070_at

Cbx3 chromobox homolog 3 (Drosophila HP1 gamma) chr1 1435135_at

Ccdc30 coiled-coil domain containing 30 chr4 1435236_at

Ccdc53 coiled-coil domain containing 53 chr10 1435346_at

Ccdc82 coiled-coil domain containing 82 chr9_random 1435452_at

Ccnd2 cyclin D2 chr6 1435471_at

1435474_at

Cct6a chaperonin containing Tcp1, subunit 6a (zeta) chr5 1435487_at

Chmp4b chromatin modifying protein 4B chr2 1435545_at

Cldn9 claudin 9 chr17 1435642_at

Commd5 COMM domain containing 5 chr15 1435663_at

Comp cartilage oligomeric matrix protein chr8 1435693_at

Csf1r colony stimulating factor 1 receptor chr18 1435753_a_at

Ctla4 cytotoxic T-lymphocyte-associated protein 4 chr1 1435867_at

Cts3 cathepsin 3 chr13 1435921_at

Ctsl cathepsin L chr13 1435948_at

Cul7 cullin 7 chr17 1436004_at

Ddx3y DEAD (Asp-Glu-Ala-Asp) box polypeptide 3, Y-linked chrY 1436196_at

1436283_at

1436478_at

Defa-rs1 defensin, alpha, related sequence 1 chr8 1436555_at

Derl1 Der1-like domain family, member 1 chr15 1436630_at

Dkk4 dickkopf homolog 4 (Xenopus laevis) chr8 1436705_at

Dnpep aspartyl aminopeptidase chr1 1436732_s_at

Dync1i1 dynein cytoplasmic 1 intermediate chain 1 chr6 1436739_at

Ebf3 early B-cell factor 3 chr7 1436982_at

Egr2 early growth response 2 chr10 1437019_at

Eif2s3y eukaryotic translation initiation factor 2, subunit 3, chrY 1437069_at

structural gene Y-linked

Evi2b ecotropic viral integration site 2b chr11 1437130_at

Fam115a family with sequence similarity 115, member A chr6 1437193_s_at

Fam190a family with sequence similarity 190, member A chr6 1437222_x_at

Fezf2 Fez family zinc finger 2 chr14 1437353_at

Flrt3 fibronectin leucine rich transmembrane protein 3 chr2 1437360_at

1437517_x_at

Foxp2 forkhead box P2 chr6 1437542_at

1437550_at

Frk fyn-related kinase chr10 1437601_at

1437745_at

Ggnbp2 gametogenetin binding protein 2 chr11 1437857_at

Gli3 GLI-Kruppel family member GLI3 chr13 1437858_at

Glul glutamate-ammonia ligase (glutamine synthetase) chr1 1437996_s_at

Speer4b spermatogenesis associated glutamate (E)-rich protein 4b chr5 1438015_at

Gpd2 glycerol phosphate dehydrogenase 2, mitochondria chr2 1438139_at

1438232_at

Gpx1 glutathione peroxidase 1 chr9 1438247_at

Grb14 growth factor receptor bound protein 14 chr2 1438311_at

Grid2 "glutamate receptor, ionotropic, delta 2" chr6 1438396_at

1438433_at

Hbegf heparin-binding EGF-like growth factor chr18 1438445_at

Hoxd8 homeobox D8 chr2 1438585_at

Hpse heparanase chr5 1438683_at

Hsd17b10 hydroxysteroid (17-beta) dehydrogenase 10 chrX 1438725_at

Hspb1 heat shock protein 1 chr5 1438774_s_at

1438787_at

Iah1 isoamyl acetate-hydrolyzing esterase 1 homolog (S. cerevisiae) chr12 1439010_at

Il23a interleukin 23, alpha subunit p19 chr10 1439059_at

Irx1 Iroquois related homeobox 1 (Drosophila) chr13 1439127_at

Irx3 Iroquois related homeobox 3 (Drosophila) chr8 1439131_at

Itm2a integral membrane protein 2A chrX 1439207_at

Kcnj13 potassium inwardly-rectifying channel, subfamily J, member 13 chr1 1439237_a_at

Kdm5b lysine (K)-specific demethylase 5B chr1 1439338_at

1439387_x_at

Klhl22 kelch-like 22 (Drosophila) chr16 1439427_at

Krt25 keratin 25 chr11 1439487_at

Ldb2 LIM domain binding 2 chr5 1439501_at

Ldhc lactate dehydrogenase C chr7 1439565_at

1439694_at

Lias lipoic acid synthetase chr5 1439730_at

Mup major urinary protein chr4 1439836_at

Lor loricrin chr3 1439958_at

Lrrc6 leucine rich repeat containing 6 (testis) chr15 1439991_a_at

Lrrtm1 leucine rich repeat transmembrane neuronal 1 chr6 1440046_at

Lypd6b LY6/PLAUR domain containing 6B chr2 1440108_at

Maf1 MAF1 homolog (S. cerevisiae) chr15 1440199_at

1440542_at

Mall mal, T-cell differentiation protein-like chr2 1440715_s_at

Marcksl1 MARCKS-like 1 chr4 1440738_at

Mobkl3 MOB1, Mps One Binder kinase activator-like 3 (yeast) chr1 1440870_at

Muc15 mucin 15 chr2 1440991_at

Myot myotilin chr18 1441026_at

Nde1 nuclear distribution gene E homolog 1 (A nidulans) chr16 1441031_at

Nelf nasal embryonic LHRH factor chr2 1441428_at

Nfatc1 nuclear factor of activated T-cells, cytoplasmic, chr18 1441534_at

calcineurin-dependent 1

Npas3 neuronal PAS domain protein 3 chr12 1441708_at

Olfml3 olfactomedin-like 3 chr3 1441788_s_at

Ostm1 osteopetrosis associated transmembrane protein 1 chr10 1441918_x_at

Otx1 orthodenticle homolog 1 (Drosophila) chr11 1441982_at

Oxr1 oxidation resistance 1 chr15 1442051_at

Pcdh19 protocadherin 19 chrX 1442063_at

Pcolce2 procollagen C-endopeptidase enhancer 2 chr9 1442081_at

Pcp4 Purkinje cell protein 4 chr16 1442175_at

Pde1a phosphodiesterase 1A, calmodulin-dependent chr2 1442645_at

Phlda2 pleckstrin homology-like domain, family A, member 2 chr7 1442659_at

Pip4k2a phosphatidylinositol-5-phosphate 4-kinase, type II, alpha chr2 1442891_at

Pkp2 plakophilin 2 chr16 1443552_at

Pldn pallidin chr2 1443564_at

Plin2 perilipin 2 chr4 1443843_x_at

Ppp3ca protein phosphatase 3, catalytic subunit, alpha isoform chr3 1443936_at

Prkag2 protein kinase, AMP-activated, gamma 2 non-catalytic subunit chr5 1443943_at

Prkar2a protein kinase, cAMP dependent regulatory, type II alpha chr9 1443966_at

Prl8a2 prolactin family 8, subfamily a, member 2 chr13 1444004_at

Prnp prion protein chr2 1444038_at

Rab3d RAB3D, member RAS oncogene family chr9 1444157_a_at

Rabgap1l RAB GTPase activating protein 1-like chr1 1444158_at

Ran RAN, member RAS oncogene family chr5 1444228_s_at

Rilpl1 Rab interacting lysosomal protein-like 1 chr5 1444389_at

Ripply3 ripply3 homolog (zebrafish) chr16 1444441_at

Rpl17 ribosomal protein L17 chr18 1444921_at

Rpl9 ribosomal protein L9 chr13 1444927_at

Rpl9 Ribosomal protein L9 chr14 1445275_at

Rusc1 RUN and SH3 domain containing 1 chr3 1446033_at

1446370_at

Sap18 Sin3-associated polypeptide 18 chr14 1446845_at

Scd2 stearoyl-Coenzyme A desaturase 2 chr19 1447027_s_at

Sdc2 syndecan 2 chr15 1447085_s_at

Serpinb3a serine (or cysteine) peptidase inhibitor, clade B (ovalbumin), chr1 1447097_at

member 3A

Serpinb6e serine (or cysteine) peptidase inhibitor, clade B, member 6e chr13 1447292_at

Sesn1 sestrin 1 chr10 1447643_x_at

Sgk3 serum/glucocorticoid regulated kinase 3 chr1 1447667_x_at

Shox2 short stature homeobox 2 chr3 1447707_s_at

Sirpb1a,1b signal-regulatory protein beta 1-like, 1A, 1B chr3 1439767_at

Slc37a4 solute carrier family 37 (glucose-6-phosphate transporter), member 4 chr9 1447729_s_at

Slco1a4 solute carrier organic anion transporter family, member 1a4 chr6 1447757_x_at

Snai2 snail homolog 2 (Drosophila) chr16 1447800_x_at

Snrpb small nuclear ribonucleoprotein B chr2 1447842_x_at

Specc1 sperm antigen with calponin homology and coiled-coil domains 1 chr11 1447895_x_at

Speer4a spermatogenesis associated glutamate (E)-rich protein 4a chr5 1447931_at

Srgap1 SLIT-ROBO Rho GTPase activating protein 1 chr10 1434979_at

St5 suppression of tumorigenicity 5 chr7 1447936_at

St8sia1 ST8 alpha-N-acetyl-neuraminide alpha-2,8-sialyltransferase 1 chr6 1448025_at

Stx19 syntaxin 19 chr16 1452759_s_at

Tceal8 transcription elongation factor A (SII)-like 8 chrX 1452848_at

Tekt2 tektin 2 chr4 1452983_at

Tex19.2 testis expressed gene 19.2 chr11 1453028_at

Thbs4 thrombospondin 4 chr13 1453032_at

Tmem27 transmembrane protein 27 chrX 1453102_at

Tmigd1 transmembrane and immunoglobulin domain containing 1 chr11 1453119_at

Tmx4 thioredoxin-related transmembrane protein 4 chr2 1453120_at

Tnfsf10 tumor necrosis factor (ligand) superfamily, member 10 chr3 1453365_at

Tor3a torsin family 3, member A chr1 1453581_at

Tpd52l1 tumor protein D52-like 1 chr10 1454095_at

Tram1l1 translocation associated membrane protein 1-like 1 chr3 1454354_at

Trim6 tripartite motif-containing 6 chr7 1454671_at

Tspan8 tetraspanin 8 chr10 1454680_at

Tusc2 tumor suppressor candidate 2 chr9 1454724_x_at

Uap1l1 UDP-N-acteylglucosamine pyrophosphorylase 1-like 1 chr2 1454734_at

Ube1y1 ubiquitin-activating enzyme E1, Chr Y 1 chrY 1454857_at

Ube1y1 1454896_at

Ugt2b38 UDP glucuronosyltransferase 2 family, polypeptide B38 chr5 1454990_at

Uts2r urotensin 2 receptor chr11 1455111_at

Vasn vasorin chr16 1455135_at

Vav3 vav 3 oncogene chr3 1455154_at

1455159_at

Vcl vinculin chr14 1455227_at

Vldlr very low density lipoprotein receptor chr19 1455507_s_at

Wbp5 WW domain binding protein 5 chrX 1455586_at

Zfy1/2 zinc finger protein 2, Y linked chrY 1455718_at

2410004A20Rik RIKEN cDNA 2410004A20 gene chr9 1433542_at

4930579H20Rik RIKEN cDNA 4930579H20 gene chr10 1433701_at

8030476L19Rik RIKEN cDNA 8030476L19 gene chr15 1433771_at

D630042F21Rik RIKEN cDNA D630042F21 gene chr6 1436085_at

Gm10839 predicted gene 10839 chr15 1438028_at

Gm4814 predicted gene 4814 chr13 1438042_at

LOC639910 hypothetical protein LOC639910 random 1439855_at
