# Supplementary material for: The Presence of the Y-Chromosome, Not the Absence of the Second X-Chromosome, Alters the mRNA Levels Stored in the Fully Grown XY Mouse Oocyte
Source: PLoS One. 2012 Jul 6;7(7):e40481. doi: 10.1371/journal.pone.0040481 (PMC3391287; doi:10.1371/journal.pone.0040481)
Supplement: Table S2 — Differentially expressed genes at lower levels in XY oocytes than in XX oocytes, identified by cDNA microarray. This table provides the list of genes which were found to be expressed at lower levels in XY oocytes than in XX oocytes by at least 2-fold (p<0.05, students t-test). (DOC) [file pone.0040481.s002.doc]

Table S2. Differentially expressed genes identified by cDNA microarray analysis

**Lower in XY oocytes than in XX oocytes by at least 2-fold**

Gene symbol Gene name Chromosome Probe set ID

Abcb7 ATP-binding cassette, sub-family B (MDR/TAP), member 7 chrX 1427937_at

Abcd1 ATP-binding cassette, sub-family D (ALD), member 1 chrX 1455424_at

Abhd13 abhydrolase domain containing 13 chr8 1450071_at

Acbd3 acyl-Coenzyme A binding domain containing 3 chr1 1418501_a_at

Actb actin, beta chr5 1422901_at

Acvr1b activin A receptor, type 1B chr15 1419527_at

Adam10 a disintegrin and metallopeptidase domain 10 chr9 1420817_at

Adam12 a disintegrin and metallopeptidase domain 12 (meltrin alpha) chr17 1426123_a_at

Adam17 a disintegrin and metallopeptidase domain 17 chr12 1460214_at

Adamtsl1 ADAMTS-like 1 chr4 1417588_at

Add2 adducin 2 (beta) chr6 1429993_s_at

Adnp2 ADNP homeobox 2 chr18 1430271_x_at

Aebp2 AE binding protein 2 chr6 1420006_at

Aff3 AF4/FMR2 family, member 3 chr1 1417400_at

Ahr aryl-hydrocarbon receptor chr12 1420946_at

Ahsa2 AHA1, activator of heat shock protein ATPase homolog 2 (yeast) chr11 1435160_at

Aig1 androgen-induced 1 chr10 1453865_a_at

Ak2 adenylate kinase 2 chr4 1427261_at

Ak5 adenylate kinase 5 chr3 1415822_at

Ampd2 adenosine monophosphate deaminase 2 chr3 1429152_at

Appl1 adaptor protein, phosphotyrosine interaction, chr14 1420989_at

PH domain and leucine zipper containing 1

Aqp9 aquaporin 9 chr9 1417168_a_at

Arhgap28 Rho GTPase activating protein 28 chr17 1418173_at

Arid2 AT rich interactive domain 2 (ARID, RFX-like) chr15 1429932_at

Arid5b AT rich interactive domain 5B (MRF1-like) chr10 1423821_at

1448354_at

Asah1 N-acylsphingosine amidohydrolase 1 chr8 1430781_at

Asb15 ankyrin repeat and SOCS box-containing 15 chr6 1417169_at

Ash1l ash1 (absent, small, or homeotic)-like (Drosophila) chr3 1438391_x_at

1450285_at

Atp2b3 ATPase, Ca++ transporting, plasma membrane 3 chrX 1427964_at

Atp2c2 ATPase, Ca++ transporting, type 2C, member 2 chr8 1450906_at

Atp7a ATPase, Cu++ transporting, alpha polypeptide chrX 1449260_at

Atrnl1 attractin like 1 chr19 1434465_x_at

Atrx alpha thalassemia/mental retardation syndrome X-linked homolog chrX 1417434_at

1418155_at

1420973_at

1448600_s_at

Atxn1l ataxin 1-like chr8 1452093_at

1428600_at

Axin2 axin2 chr11 1422943_a_at

B3gnt2 /// Commd1 UDP-GlcNAc:betaGal beta-1,3-N-acetylglucosaminyltransferase 2 chr11 1417837_at

/// COMM domain containing 1 1419498_at

Baiap2l1 BAI1-associated protein 2-like 1 chr5 1422660_at

Bcar1 breast cancer anti-estrogen resistance 1 chr8 1450449_a_at

Bmp15 bone morphogenetic protein 15 chrX 1416131_s_at

1419455_at

Brwd3 bromodomain and WD repeat domain containing 3 chrX 1430776_s_at

Btbd1 BTB (POZ) domain containing 1 chr2 1417593_at

C1galt1c1 C1GALT1-specific chaperone 1 chrX 1426401_at

Cachd1 cache domain containing 1 chr4 1422021_at

Cand1 cullin associated and neddylation disassociated 1 chr10 1460671_at

Cbara1 calcium binding atopy-related autoantigen 1 chr10 1416221_at

Cbl Casitas B-lineage lymphoma chr6 1438545_at

Ccdc22 coiled-coil domain containing 22 1430435_at

Ccnj cyclin J chr19 1422631_at

Cdk16 cyclin-dependent kinase 16 chrX 1433537_at

Cdkn1b cyclin-dependent kinase inhibitor 1B chr6 1429320_at

Cdkn2aip CDKN2A interacting protein chr8 1418517_at

Cdkn2aipnl CDKN2A interacting protein N-terminal like chr11 1424471_at

Cdv3 carnitine deficiency-associated gene expressed in ventricle 3 chr14 1437742_at

Cdyl chromodomain protein, Y chromosome-like chr13 1419459_a_at

Celf5 CUGBP, Elav-like family member 5 chr10 1455683_a_at

Cep170 centrosomal protein 170 chr1 1426478_at

Cep57 centrosomal protein 57 chr9 1451230_a_at

Cetn2 centrin 2 chrX 1425216_at

1451268_at

Cgnl1 cingulin-like 1 chr9 1430190_at

Chd4 chromodomain helicase DNA binding protein 4 chr6 1438695_at

Chd7 chromodomain helicase DNA binding protein 7 chr4 1450394_at

Chdh choline dehydrogenase chr14 1419529_at

Chmp1b chromatin modifying protein 1B chr18 1436859_at

Chst11 carbohydrate sulfotransferase 11 chr10 1415846_a_at

Ckb creatine kinase, brain chr4 1427739_a_at

Clcn5 chloride channel 5 chrX 1421106_at

1448595_a_at

Cmtm8 CKLF-like MARVEL transmembrane domain containing 8 chr9 1422900_at

Cnot6 CCR4-NOT transcription complex, subunit 6 chr11 1440936_at

Cnpy1 canopy 1 homolog (zebrafish) chr5 1418579_at

Commd9 COMM domain containing 9 chr2 1429061_at

1428334_at

Cpox coproporphyrinogen oxidase chr16 1451126_at

Crkl v-crk sarcoma virus CT10 oncogene homolog (avian)-like chr16 1433540_x_at

Crls1 cardiolipin synthase 1 chr2 1437103_at

Crnkl1 Crn, crooked neck-like 1 (Drosophila) chr2 1425516_at

Csf1 colony stimulating factor 1 (macrophage) chr3 1435333_at

Csnk2a2 casein kinase 2, alpha prime polypeptide chr8 1460737_at

Ctbs chitobiase, di-N-acetyl- chr3 1431107_at

1452869_at

Ctsh cathepsin H chr9 1455812_x_at

Ctu2 cytosolic thiouridylase subunit 2 homolog (S. pombe) chr8 1429633_at

Cul4b cullin 4B chrX 1448908_at

Cul5 cullin 5 chr9 1427543_s_at

Cxcr3 chemokine (C-X-C motif) receptor 3 chrX 1417831_at

Cyp4f39 cytochrome P450, family 4, subfamily f, polypeptide 39 chr17 1419922_s_at

Cyp7b1 cytochrome P450, family 7, subfamily b, polypeptide 1 chr3 1431231_at

1452504_s_at

Dbf4 DBF4 homolog (S. cerevisiae) chr5 1454673_at

Dcpp3 demilune cell and parotid protein 3 chr17 1450710_at

Ddx3x DEAD/H (Asp-Glu-Ala-Asp/His) box polypeptide 3, X-linked chrX 1420849_at

1448468_a_at

1449578_at

Ddx5 DEAD (Asp-Glu-Ala-Asp) box polypeptide 5 chr11 1448772_at

Ddx6 DEAD (Asp-Glu-Ala-Asp) box polypeptide 6 chr2 1425447_at

Dhx36 DEAH (Asp-Glu-Ala-His) box polypeptide 36 chr3 1419279_at

Dkc1 dyskeratosis congenita 1, dyskerin homolog (human) chrX 1426438_at

1426558_x_at

Dlgap2 discs, large (Drosophila) homolog-associated protein 2 chr8 1425964_x_at

Dnajb4 DnaJ (Hsp40) homolog, subfamily B, member 4 chr3 1451539_at

Dnajc3 DnaJ (Hsp40) homolog, subfamily C, member 3 chr14 1429682_at

Dnmt3a DNA methyltransferase 3A chr12 1451273_x_at

Dpy19l3 dpy-19-like 3 (C. elegans) chr7 1428194_at

1431930_x_at

Dusp7 dual specificity phosphatase 7 chr2 1434518_at

Eif1ax eukaryotic translation initiation factor 1A, X-linked chrX 1441182_at

Eif2s2 eukaryotic translation initiation factor 2, subunit 2 (beta) chr2 1428193_at

Eif5b eukaryotic translation initiation factor 5B chr1 1422726_x_at

Esco1 establishment of cohesion 1 homolog 1 (S. cerevisiae) chr18 1420412_at

Esr1 estrogen receptor 1 (alpha) chr10 1424685_at

Extl3 exostoses (multiple)-like 3 chr14 1421075_s_at

F8a factor 8-associated gene A chrX 1452471_at

Fbxl14 F-box and leucine-rich repeat protein 14 chr6 1455653_at

Fbxo5 F-box protein 5 chr10 1450775_at

Fbxw16, 23 F-box and WD-40 domain protein 16, 23 chr9 1450039_at

Fbxw8 F-box and WD-40 domain protein 8 chr5 1424645_at

Fermt2 fermitin family homolog 2 (Drosophila) chr14 1437226_x_at

Ffar2 free fatty acid receptor 2 chr7 1450044_at

1450105_at

Fignl1 fidgetin-like 1 chr11 1429046_at

Foxo3 forkhead box O3 chr10 1455106_a_at

Frmd4a FERM domain containing 4A chr2 1450038_s_at

Fstl1 follistatin-like 1 chr16 1416917_at

Ftsjd1 FtsJ methyltransferase domain containing 1 chr8 1419108_at

1420700_s_at

Fzd3 frizzled homolog 3 (Drosophila) chr14 1456190_a_at

Fzd7 frizzled homolog 7 (Drosophila) chr1 1421840_at

G6pdx glucose-6-phosphate dehydrogenase X-linked chrX 1451140_s_at

Galnt3 UDP-N-acetyl-alpha-D-galactosamine:polypeptide N- chr2 1430304_at

acetylgalactosaminyltransferase 3

Gimap8 GTPase, IMAP family member 8 chr6 1419872_at

Gja4 gap junction protein, alpha 4 chr4 1435405_at

Golph3 golgi phosphoprotein 3 chr15 1425290_at

Gosr1 golgi SNAP receptor complex member 1 chr11 1433442_at

Gpbp1 GC-rich promoter binding protein 1 chr13 1448116_at

Gpr137b G protein-coupled receptor 137B chr13 1423889_at

1452614_at

Gpr137b, 137b-ps G protein-coupled receptor 137B chr13 1450210_at

Gpr137b-ps G protein-coupled receptor 137B, pseudogene chr13 1451369_at

Gpr137c G protein-coupled receptor 137C chr14 1429499_at

Gsg2 germ cell-specific gene 2 chr11 1452326_at

Gspt2 G1 to S phase transition 2 chrX 1418790_at

Gtpbp10 GTP-binding protein 10 (putative) chr5 1427142_s_at

Gyk glycerol kinase chrX 1451640_a_at

H2-Aa histocompatibility 2, class II antigen A, alpha chr17 1420459_at

Heca headcase homolog (Drosophila) chr10 1420405_at

1422648_at

Hectd2 HECT domain containing 2 chr19 1417042_at

Herc2 hect (homologous to the E6-AP (UBE3A) carboxyl terminus) domain chr7 1418367_x_at

and RCC1 (CHC1)-like domain (RLD) 2

Hif1a hypoxia inducible factor 1, alpha subunit chr12 1416735_at

Hist1h3f histone cluster 1, H3f chr13 1431809_at

Hist1h4a/4b/4f/4m histone cluster 1, H4a, H4b, H4f , H4m chr13 1422837_at

Hist2h2aa1/2aa2/2ac/3c1

histone cluster 2, H2aa1, H2aa2, H2ac, H3c1" chr3 1426570_a_at

Hist2h3c1 histone cluster 2, H3c1 chr3 1451100_a_at

Hjurp Holliday junction recognition protein chr1 1438625_s_at

Hmga2 high mobility group AT-hook 2 chr1 1427253_s_at

1438040_a_at

Hnrnpa2b1 heterogeneous nuclear ribonucleoprotein A2/B1 chr6 1449925_at

Hnrnpa3 heterogeneous nuclear ribonucleoprotein A3 chr2 1423645_a_at

Homer1 homer homolog 1 (Drosophila) chr13 1420465_s_at

Hsp90b1 heat shock protein 90, beta (Grp94), member 1 chr10 1416645_a_at

1421096_at

Hspa1b heat shock protein 1B chr17 1427416_x_at

Hspa5 heat shock protein 5 chr2 1433579_at

Huwe1 HECT, UBA and WWE domain containing 1 chrX 1452078_a_at

Igbp1 immunoglobulin (CD79A) binding protein 1 chrX 1418898_at

1428372_at

1431785_at

Igf2bp2 insulin-like growth factor 2 mRNA binding protein 2 chr16 1426481_at

Igf2bp3 insulin-like growth factor 2 mRNA binding protein 3 chr6 1417122_at

1460337_at

Igh-3, Ighg immunoglobulin heavy chain 3 (serum IgG2b), (gamma polypeptide) chr12 1416154_at

Ighg Immunoglobulin heavy chain (gamma polypeptide) chr12 1418550_x_at

Ikzf2 IKAROS family zinc finger 2 chr1 1449368_at

Il10rb interleukin 10 receptor, beta chr16 1429362_a_at

Il17rd interleukin 17 receptor D chr14 1437991_x_at

Inpp5f inositol polyphosphate-5-phosphatase F chr7 1415811_at

1417504_at

Insig1 insulin induced gene 1 chr5 1435064_a_at

Insr insulin receptor chr8 1448545_at

Jag1 jagged 1 chr2 1422430_at

Jak2 Janus kinase 2 chr19 1428762_at

Jam2 junction adhesion molecule 2 chr16 1424752_x_at

Jarid2 jumonji, AT rich interactive domain 2 chr13 1452364_at

Jhdm1d jumonji C domain-containing histone demethylase 1 homolog D chr6 1417039_a_at

(S. cerevisiae) 1421074_at

1433999_at

Kcnab1 K voltage-gated channel, shaker-related subfamily, beta member 1 chr3 1434888_a_at

Kdm5c lysine (K)-specific demethylase 5C chrX 1421339_at

1425671_at

Khdc1b KH domain containing 1B chr1 1455291_s_at

Khdrbs1 KH domain containing, RNA binding, signal transduction associated 1 chr4 1433887_at

Klhl15 kelch-like 15 (Drosophila) chrX 1420852_a_at

Klhl9 kelch-like 9 (Drosophila) chr4 1428349_s_at

Kras v-Ki-ras2 Kirsten rat sarcoma viral oncogene homolog chr6 1427464_s_at

Krt12 keratin 12 chr11 1450051_at

Lamp2 lysosomal-associated membrane protein 2 chrX 1455727_at

Larp4 La ribonucleoprotein domain family, member 4 chr15 1429491_s_at

Lbx1 ladybird homeobox homolog 1 (Drosophila) chr19 1429359_s_at

Lef1 lymphoid enhancer binding factor 1 chr3 1436362_x_at

Lig4 ligase IV, DNA, ATP-dependent chr8 1449670_x_at

Lin7c lin-7 homolog C (C. elegans) chr2 1419443_at

1420843_at

1453030_at

Lrrc8e leucine rich repeat containing 8 family, member E chr8 1448904_at

Mab21l1 mab-21-like 1 (C. elegans) chr3 1421172_at

Macrod1 MACRO domain containing 1 chr19 1429943_at

Mageh1 melanoma antigen, family H, 1 chrX 1449445_x_at

Magt1 magnesium transporter 1 chrX 1455988_a_at

Map3k4 mitogen-activated protein kinase kinase kinase 4 chr17 1429057_at

Map3k5 mitogen-activated protein kinase kinase kinase 5 chr10 1435290_x_at

Matr3 matrin 3 chr18 1427490_at

1438647_x_at

Mbd3l2 methyl-CpG binding domain protein 3-like 2 chr9 1425565_at

Mbtd1 mbt domain containing 1 chr11 1426236_a_at

Med13 mediator complex subunit 13 chr11 1448256_at

Mfap1a microfibrillar-associated protein 1A chr2 1420846_at

1426685_a_at

Mgea5 meningioma expressed antigen 5 (hyaluronidase) chr19 1416361_a_at

1421052_a_at

1426957_at

Mia3 melanoma inhibitory activity 3 chr1_random 1420183_at

Micall2 MICAL-like 2 chr5 1433725_at

Mllt11 myeloid/lymphoid or mixed-lineage leukemia; translocated to, 11 chr3 1431076_at

Mmgt1 membrane magnesium transporter 1 chrX 1438644_x_at

Mon1b MON1 homolog b (yeast) chr8 1454686_at

Morc3 microrchidia 3 chr16 1429486_at

Morf4l1 mortality factor 4 like 1 chr9 1429625_at

Mos Moloney sarcoma oncogene chr4 1451728_at

Mospd1 motile sperm domain containing 1 chr1 1421094_at

Mpped1 metallophosphoesterase domain containing 1 chr15 1420171_s_at

Mrps2 mitochondrial ribosomal protein S2 chr2 1452664_a_at

Msl2 male-specific lethal 2 homolog (Drosophila) chr9 1416127_a_at

1418170_a_at

1427414_at

Myh9 myosin, heavy polypeptide 9, non-muscle chr15 1426439_at

N4bp2 NEDD4 binding protein 2 chr5 1422703_at

Naa16 N(alpha)-acetyltransferase 16, NatA auxiliary subunit chr14 1422902_s_at

Nceh1 arylacetamide deacetylase-like 1 chr3 1421750_a_at

1448504_a_at

Ndufaf4 NADH dehydrogenase (ubiquinone) 1 alpha subcomplex, chr4 1426448_at

assembly factor 4

Nfkbie nuclear factor of kappa light polypeptide gene enhancer chr17 1419049_at

in B-cells inhibitor, epsilon

Nin ninein chr12 1438360_x_at

Nkrf NF-kappaB repressing factor chrX 1419687_at

Nlk nemo like kinase chr11 1429310_at

Nucks1 nuclear casein kinase and cyclin-dependent kinase substrate 1 chr1 1428512_at

Ocrl oculocerebrorenal syndrome of Lowe chrX 1416207_at

Ofd1 oral-facial-digital syndrome 1 gene homolog (human) chrX 1437992_x_at

Ogt O-linked N-acetylglucosamine (GlcNAc) transferase (UDP-N- chrX 1415847_at

acetylglucosamine:polypeptide-N-acetylglucosaminyl transferase) 1415889_a_at

1431099_at

Olfm1 olfactomedin 1 chr2 1428146_s_at

1437533_at

Olfr288 olfactory receptor 288 chr15 1450886_at

Olfr976 olfactory receptor 976 chr9 1429274_at

Ophn1 oligophrenin 1 chrX 1433829_a_at

Otud1 OTU domain containing 1 chr2 1424598_at

Otud5 OTU domain containing 5 chrX 1427936_at

Pabpn1l poly(A)binding protein nuclear 1-like chr8 1418628_at

Pan3 PAN3 polyA specific ribonuclease subunit homolog (S. cerevisiae) chr5 1429591_at

Parp4 poly (ADP-ribose) polymerase family, member 4 chr14 1418838_at

Pcdh9 protocadherin 9 chr14 1451003_at

Pcnx pecanex homolog (Drosophila) chr12 1430981_s_at

Pde2a phosphodiesterase 2A, cGMP-stimulated chr7 1420021_s_at

Pdgfb platelet derived growth factor, B polypeptide chr15 1439255_s_at

Pdik1l PDLIM1 interacting kinase 1 like chr4 1435737_a_at

Pfkfb2 6-phosphofructo-2-kinase/fructose-2,6-biphosphatase 2 chr1 1435133_at

Pfn1 profilin 1 chr11 1417673_at

Pgm2l1 phosphoglucomutase 2-like 1 chr18 1453752_at

Phf8 PHD finger protein 8 chrX 1422045_a_at

Phka2 phosphorylase kinase alpha 2 chrX 1451983_at

Piga phosphatidylinositol glycan anchor biosynthesis, class A chrX 1426895_at

Pja1 praja1, RING-H2 motif containing chrX 1426941_at

1437175_at

Plag1 pleiomorphic adenoma gene 1 chr4 1419695_at

Plagl2 pleiomorphic adenoma gene-like 2 chr2 1431695_at

Plekhg1 pleckstrin homology domain containing, chr10 1415790_at

family G (with RhoGef domain) member 1

Plxnc1 plexin C1 chr10 1426562_a_at

Pnma5 paraneoplastic antigen family 5 chrX 1427068_x_at

Ppap2b phosphatidic acid phosphatase type 2B chr4 1427143_at

Ppfibp1 PTPRF interacting protein, binding protein 1 (liprin beta 1) chr6 1427333_s_at

Ppp1cb protein phosphatase 1, catalytic subunit, beta isoform chr5 1457776_at

Ppp1r14b protein phosphatase 1, regulatory (inhibitor) subunit 14B chr19 1455642_a_at

Prdm16 PR domain containing 16 chr4 1427334_s_at

Prpf38b PRP38 pre-mRNA processing factor 38 (yeast) domain containing B chr3 1450923_at

Prr18 proline rich region 18 chr17 1450135_at

Prr24 proline rich 24 chr7 1430582_at

Prss44 protease, serine, 44 chr9 1434000_at

Ptcd1 pentatricopeptide repeat domain 1 chr5 1417696_at

Ptpn12 protein tyrosine phosphatase, non-receptor type 12 chr5 1451419_at

Ptprf protein tyrosine phosphatase, receptor type, F chr4 1427127_x_at

1448183_a_at

Ptprg protein tyrosine phosphatase, receptor type, G chr14 1450874_at

Pwwp2a PWWP domain containing 2A chr11 1416156_at

Rab21 RAB21, member RAS oncogene family chr10 1428768_at

Rab33b RAB33B, member of RAS oncogene family chr3 1436959_x_at

Rai14 retinoic acid induced 14 chr15 1431921_a_at

Rapgef3 Rap guanine nucleotide exchange factor (GEF) 3 chr15 1416055_at

1417517_at

1418180_at

Rapgefl1 Rap guanine nucleotide exchange factor (GEF)-like 1 chr11 1449711_at

Rasa1 RAS p21 protein activator 1 chr13 1418070_at

Rassf3 Ras association (RalGDS/AF-6) domain family member 3 chr10 1427430_at

Rassf5 Ras association (RalGDS/AF-6) domain family member 5 chr1 1450021_at

Rbm12 RNA binding motif protein 12 chr2 1450026_a_at

Rbm3 RNA binding motif protein 3 chr13 1437012_x_at

Rbpj recombination signal binding protein for Ig kappa J region chr5 1439256_x_at

Rdx radixin chr9 1439388_s_at

Rest RE1-silencing transcription factor chr5 1450787_at

Rfx2 regulatory factor X, 2 (influences HLA class II expression) chr17 1418484_at

Rif1 Rap1 interacting factor 1 homolog (yeast) chr2 1460331_at

Rmnd5a required for meiotic nuclear division 5 homolog A (S. cerevisiae) chr6 1451527_at

Rnaset2a ribonuclease T2A chr17 1428323_at

Rnf122 ring finger protein 122 chr8 1439433_a_at

Rnf145 ring finger protein 145 chr11 1427400_at

Rnf168 ring finger protein 168 chr16 1424649_a_at

Rnf182 ring finger protein 182 chr13 1426505_at

Rock1 Rho-associated coiled-coil containing protein kinase 1 chr18 1449978_at

Rph3al rabphilin 3A-like (without C2 domains) chr11 1452721_a_at

Rprd2 regulation of nuclear pre-mRNA domain containing 2 chr3 1456598_at

Rrbp1 ribosome binding protein 1 chr2 1429377_at

Rrm2b ribonucleotide reductase M2 B (TP53 inducible) chr13 1423397_at

Rsrc2 arginine/serine-rich coiled-coil 2 chr5 1425517_s_at

Sacs sacsin chr14 1424325_at

Scel sciellin chr14 1435228_at

Scml2 sex comb on midleg-like 2 (Drosophila) chrX 1416391_at

Seh1l SEH1-like (S. cerevisiae chr18 1436030_at

Sema5a sema domain, seven thrombospondin repeats (type 1 and type 1-like), chr15 1424470_a_at

transmembrane domain (TM), short cytoplasmic domain, (semaphorin) 5A

Serac1 serine active site containing 1 chr17 1438941_x_at

Serbp1 serpine1 mRNA binding protein 1 chr6 1431417_at

Serinc1 serine incorporator 1 chr10 1453996_a_at

Setd1b SET domain containing 1B chr5 1451863_at

Setd4 SET domain containing 4 chr16 1433456_at

Sf3b2 splicing factor 3b, subunit 2 chr19 1460551_at

Sh3kbp1 SH3-domain kinase binding protein 1 chrX 1418816_at

1424679_at

Shprh SNF2 histone linker PHD RING helicase chr10 1455435_s_at

Shroom2 shroom family member 2 chrX 1454811_a_at

Shroom4 shroom family member 4 chrX 1427912_at

Slc11a2 solute carrier family 11 (divalent metal ion transporters), member 2 chr15 1449262_s_at

Slc12a2 solute carrier family 12, member 2 chr18 1418774_a_at

1419736_a_at

Slc12a6 solute carrier family 12, member 6 chr2 1421436_at

Slc17a2 solute carrier family 17 (sodium phosphate), member 2 chr13 1419370_a_at

Slc25a48 solute carrier family 25, member 48 chr13 1453099_at

Slc25a5 solute carrier family 25 (mitochondrial carrier, adenine nucleotide chr5 1434163_at

translocator), member 5 1437723_s_at

Slc35a2 solute carrier family 35 (UDP-galactose transporter), member A2 chrX 1423445_at

Slc7a11 solute carrier family 7 (cationic amino acid transporter), member 11 chr3 1460274_at

Slc7a2 solute carrier family 7 (cationic amino acid transporter), member 2 chr8 1418109_at

1418986_a_at

1422851_at

Slk STE20-like kinase (yeast) chr19 1428902_at

Smc1a structural maintenance of chromosomes 1A chrX 1449018_at

Smc4 structural maintenance of chromosomes 4 chr3 1429400_at

Smg1 SMG1 homolog, PI3-kinase-related kinase (C. elegans) chr15 1421065_at

Sms spermine synthase chr3 1423169_at

Smurf2 SMAD specific E3 ubiquitin protein ligase 2 chr11 1424631_a_at

Snhg6 small nucleolar RNA host gene (non-protein coding) 6 chr1 1431818_at

Soat1 sterol O-acyltransferase 1 chr1 1420985_at

Sp1 trans-acting transcription factor 1 chr15 1452644_at

Spag16 sperm associated antigen 16 chr1 1425020_at

Spata21 spermatogenesis associated 21 chr4 1454802_x_at

Spry1 sprouty homolog 1 (Drosophila) chr3 1448233_at

Spry4 sprouty homolog 4 (Drosophila) chr18 1416255_at

Spsb4 splA/ryanodine receptor domain and SOCS box containing 4 chr9 1450781_at

Srp54a, 54b signal recognition particle 54A, 54B chr12 1421340_at

Srsf15 serine/arginine-rich splicing factor 15 chr16 1455883_a_at

Ssh1 slingshot homolog 1 (Drosophila) chr5 1454781_x_at

Stag1 stromal antigen 1 chr9 1422947_at

Stk35 serine/threonine kinase 35 chr2 1421101_a_at

Supt16h suppressor of Ty 16 homolog (S. cerevisiae) chr14 1421998_at

Suz12 suppressor of zeste 12 homolog (Drosophila) chr11 1416408_at

1423608_at

1427969_s_at

1457810_at

Syne2 synaptic nuclear envelope 2 chr12 1417210_at

Tab2 TGF-beta activated kinase 1/MAP3K7 binding protein 2 chr10 1421341_at

Tab3 TGF-beta activated kinase 1/MAP3K7 binding protein 3 chrX 1434605_at

Tacc1 transforming, acidic coiled-coil containing protein 1 chr8 1451177_at

Taf1d TATA box binding protein-associated factor, RNA polymerase I, D chr9 1429138_at

Taf5 TAF5 RNA polymerase II, TATA box binding protein-associated factor chr19 1434503_s_at

Taf7 TAF7 RNA polymerase II, TATA box binding protein)-associated factor chr18 1415949_at

1424201_a_at

1434045_at

Taz tafazzin chrX 1449252_at

Tbc1d15 TBC1 domain family, member 15 chr10 1416420_a_at

Tceanc transcription elongation factor A (SII) N-terminal and central domain chrX 1420922_at

Tcn2 transcobalamin 2 chr11 1423043_s_at

1451302_at

1452002_at

Tgfb2 transforming growth factor, beta 2 chr1 1456142_x_at

Thnsl1 threonine synthase-like 1 (bacterial) chr2 1448608_at

Thoc2 THO complex 2 chrX 1417609_at

1418350_at

1419516_at

Tm7sf3 transmembrane 7 superfamily member 3 chr6 1438931_s_at

Tmem168 transmembrane protein 168 chr6 1427305_at

Tmem181a/181b-ps transmembrane protein 181A, 181B, pseudogene chr17 1449298_a_at

1448546_at

Tmem185b transmembrane protein 185B chr1 1455956_x_at

Tmem20 transmembrane protein 20 chr19 1427172_at

Tmem30b transmembrane protein 30B chr12 1448721_at

Tmpo thymopoietin chr10 1448641_at

Tmtc1 transmembrane and tetratricopeptide repeat containing 1 chr6 1452385_at

Tnrc6b trinucleotide repeat containing 6b chr15 1448819_at

Tnrc6c trinucleotide repeat containing 6C chr11 1427276_at

Trip11 thyroid hormone receptor interactor 11 chr12 1419112_at

Trp53 transformation related protein 53 chr11 1420046_s_at

Trp53bp1 transformation related protein 53 binding protein 1 chr2 1449388_at

Trpc1 transient receptor potential cation channel, subfamily C, member 1 chr9 1436950_at

Txlng taxilin gamma chrX 1423042_at

Uba1 ubiquitin-like modifier activating enzyme 1 chrX 1423876_at

Ube2a ubiquitin-conjugating enzyme E2A, RAD6 homolog (S. cerevisiae) chrX 1420037_at

1424448_at

Ubl4 ubiquitin-like 4 chrX 1455796_x_at

Ublcp1 ubiquitin-like domain containing CTD phosphatase 1 chr11 1451310_a_at

Ubqln2 ubiquilin 2 chrX 1427359_at

Ubxn2a UBX domain protein 2A chr12 1460220_a_at

Ugcg UDP-glucose ceramide glucosyltransferase chr4 1422933_at

Uhrf1 ubiquitin-like, containing PHD and RING finger domains, 1 chr17 1423170_at

Unc5c unc-5 homolog C (C. elegans) chr3 1430229_at

Usp2 ubiquitin specific peptidase 2 chr9 1426174_s_at

1427682_a_at

Usp27x ubiquitin specific peptidase 27, X chromosome chrX 1434958_at

Usp9x ubiquitin specific peptidase 9, X chromosome chrX 1416313_at

1416487_a_at

1420679_a_at

Uxt ubiquitously expressed transcript chr18 1450914_at

Vbp1 von Hippel-Lindau binding protein 1 chrX 1438736_at

Wasf2 WAS protein family, member 2 chr4 1418365_at

1455286_at

Wdr13 WD repeat domain 13 chrX 1428274_s_at

Wdr37 WD repeat domain 37 chr13 1422492_at

Wdr76 WD repeat domain 76 chr2 1417349_at

Whamm WAS protein homolog associated with actin, chr7 1420000_s_at

golgi membranes and microtubules

Whsc1l1 Wolf-Hirschhorn syndrome candidate 1-like 1 (human) chr8 1418171_at

Wnk2 WNK lysine deficient protein kinase 2 chr13 1451202_at

Wwc1 WW, C2 and coiled-coil domain containing 1 chr11 1436506_a_at

Xiap X-linked inhibitor of apoptosis chrX 1420413_at

Xlr5a/5b/5c X-linked lymphocyte-regulated 5A, 5B, 5C chrX 1429108_at

Yipf6 Yip1 domain family, member 6 chrX 1417407_at

Ywhag tyrosine 3-monooxygenase/tryptophan 5-monooxygenase chr5 1434831_a_at

activation protein, gamma polypeptide

Zbtb33 zinc finger and BTB domain containing 33 chrX 1420660_at

1424011_at

Zbtb34 zinc finger and BTB domain containing 34 chr2 1448318_at

Zbtb37 zinc finger and BTB domain containing 37 chr1 1429119_at

Zbtb46 zinc finger and BTB domain containing 46 chr2 1428402_at

Zcchc14 zinc finger, CCHC domain containing 14 chr8 1416928_at

1421859_at

Zcchc3 zinc finger, CCHC domain containing 3 chr2 1449700_at

Zfp148 zinc finger protein 148 chr16 1436890_at

Zfp182 zinc finger protein 182 chrX 1417623_at

Zfp191 zinc finger protein 191 chr18 1418168_at

Zfp646 zinc finger protein 646 chr7 1418334_at

Zfp654 zinc finger protein 654 chr16 1425215_at

Zfp68 zinc finger protein 68 chr5 1434743_x_at

Zfp708 zinc finger protein 708 chr13 1418412_at

Zfp709 zinc finger protein 709 chr8 1437634_at

Zfp71-rs1/738 zinc finger protein 71, related sequence /// zinc finger protein 738 chr13 1429109_at

Zkscan1 zinc finger with KRAB and SCAN domains 1 chr5 1436989_s_at

Zmym3 zinc finger, MYM-type 3 chrX 1427406_at

Znrf2 zinc and ring finger 2 chr6 1426008_a_at

Zrsr2 zinc finger (CCCH type), RNA binding motif and serine/arginine rich 2 chrX 1436879_x_at

Zscan10 zinc finger and SCAN domain containing 10 chr17 1433320_at

0610010B08Rik RIKEN cDNA 0610010B08 gene, similar to zinc finger protein 14 chr2 1449799_s_at

0610010K06Rik RIKEN cDNA 0610010K06 gene chr6 1426011_a_at

1110012L19Rik RIKEN cDNA 1110012L19 gene chrX 1449068_at

1700012B15Rik RIKEN cDNA 1700012B15 gene chr12 1451988_s_at

1810013L24Rik RIKEN cDNA 1810013L24 gene chr16 1426986_at

1810063B05Rik RIKEN cDNA 1810063B05 gene chr8 1449969_at

2010002M12Rik RIKEN cDNA 2010002M12 gene chr19 1416062_at

2010002M12Rik RIKEN cDNA 2010002M12 gene 1450687_at

2200001I15Rik RIKEN cDNA 2200001I15 gene chr14 1425427_at

2210403K04Rik RIKEN cDNA 2210403K04 gene chr11 1448475_at

2410006H16Rik RIKEN cDNA 2410006H16 gene chr11 1420020_at

2610030H06Rik RIKEN cDNA 2610030H06 gene chrX 1426569_a_at

2700007P21Rik RIKEN cDNA 2700007P21 gene chr2 1424280_at

2700079J08Rik RIKEN cDNA 2700079J08 gene 1420842_at

2810474O19Rik RIKEN cDNA 2810474O19 gene chr6 1452137_at

2900054C01Rik RIKEN cDNA 2900054C01 gene chrX 1425033_at

4930519N06Rik RIKEN cDNA 4930519N06 gene chr11 1452769_at

4930566F21Rik RIKEN cDNA 4930566F21 gene chr5 1449459_s_at

4932442L08Rik RIKEN cDNA 4932442L08 gene chrX 1421066_at

4933403F05Rik RIKEN cDNA 4933403F05 gene chr18 1416655_at

1438476_a_at

4933431E20Rik RIKEN cDNA 4933431E20 gene 1451738_at

4933439F18Rik RIKEN cDNA 4933439F18 gene chr11 1452774_at

7420416P09Rik RIKEN cDNA 7420416P09 gene chr12 1449435_at

9130404H23Rik RIKEN cDNA 9130404H23 gene chr17 1455805_x_at

AI314180 expressed sequence AI314180 chr4 1419230_at

AI324046 expressed sequence AI324046 chr12 1433463_at

AI848100 expressed sequence AI848100 chr1 1420919_at

AU015836 expressed sequence AU015836 chrX 1450881_s_at

AU022751 expressed sequence AU022751 chrX 1448505_at

AU023386 expressed sequence AU023386 chr9 1417412_at

B020011L13Rik RIKEN cDNA B020011L13 gene chr1 1417332_at

BC023829 cDNA sequence BC023829 chrX 1428649_at

BC032203 cDNA sequence BC032203 chr17 1437253_at

BC052688 cDNA sequence BC052688 chr13 1432227_at

C030046E11Rik RIKEN cDNA C030046E11 gene chr19 1423064_at

C030046G05 hypothetical protein C030046G05 chr11 1418879_at

C230091D08Rik RIKEN cDNA C230091D08 gene chr7 1434180_at

C330007P06Rik RIKEN cDNA C330007P06 gene chr18 1428712_at

C86753 expressed sequence C86753 chr12 1424232_a_at

C87882 expressed sequence C87882 chr9 1452077_at

D10Bwg1379e DNA segment, Chr 10, Brigham & Women's Genetics 1379 expressed chr10 1418563_at

D11Ertd729e DNA segment, Chr 11, ERATO Doi 729, expressed chr11 1424538_at

D14Ertd426e DNA segment, Chr 14, ERATO Doi 426, expressed chr14 1417454_at

D17Wsu92e DNA segment, Chr 17, Wayne State University 92, expressed chr17 1427503_at

D1Ertd622e DNA segment, Chr 1, ERATO Doi 622, expressed chr1 1428540_at

D5Ertd579e DNA segment, Chr 5, ERATO Doi 579, expressed chr5 1447456_x_at

D9Ertd720e DNA segment, Chr 9, ERATO Doi 720, expressed chr9 1431068_at

E230008N13Rik RIKEN cDNA E230008N13 gene chr4 1422610_s_at

E330009J07Rik RIKEN cDNA E330009J07 gene chr6 1432269_a_at

E330010L02Rik RIKEN cDNA E330010L02 gene chrX 1421158_at

Fam108b family with sequence similarity 108, member B chr19 1423083_at

1460741_x_at

Fam110c family with sequence similarity 110, member C chr12 1424956_at

1429691_at

Fam116a family with sequence similarity 116, member A chr14 1416180_a_at

Fam123b family with sequence similarity 123, member B chrX 1460398_at

Fam126b family with sequence similarity 126, member B chr1 1448451_at

Fam160b1 family with sequence similarity 160, member B1 chr19 1450414_at

Fam199x family with sequence similarity 199, X-linked chrX 1415874_at

1417622_at

Fam3c family with sequence similarity 3, member C chr6 1428562_at

Fam46c family with sequence similarity 46, member C chr3 1424949_at

Fam50a family with sequence similarity 50, member A chrX 1428976_at

Fam76b family with sequence similarity 76, member B chr9 1449522_at

Fam84b family with sequence similarity 84, member B chr15 1434776_at

Gm11202 predicted gene 11202 1450937_at

Gm13032 predicted gene 13032 chr4 1429348_at

Gm16367/16427/3106/7792

predicted gene 16367, 16427, 3106, 7792 chr5 1455886_at

Gm1995/7104 REX1, RNA exonuclease 1 homolog pseudogene chr12 1416467_at

Gm4632 Predicted gene 4632 chr14 1419334_at

Gm5065 predicted gene 5065 chr7 1422638_s_at

Gm5465 predicted gene 5465 chr14 1422498_at

Gm5617 predicted gene 5617 chr9 AFFX-b-ActinMur/M12481_5_at

Gm6150 predicted gene 6150 chr10 1454884_at

Gm7969 predicted gene 7969 chr13 1420947_at

LOC100045066 hypothetical LOC100045066 chr1 1448229_s_at

LOC100503538 hypothetical LOC100503538 chr15 1452083_a_at

LOC100503807 hypothetical protein LOC100503807 chr16 1420091_s_at

LOC100504708 hypothetical LOC100504708 chrX 1449056_at
