# Supplementary material for: The Presence of the Y-Chromosome, Not the Absence of the Second X-Chromosome, Alters the mRNA Levels Stored in the Fully Grown XY Mouse Oocyte
Source: PLoS One. 2012 Jul 6;7(7):e40481. doi: 10.1371/journal.pone.0040481 (PMC3391287; doi:10.1371/journal.pone.0040481)
Supplement: Table S3 — Primer sequences. This table provides the list of primer sequences used for RT-PCR. (DOC) [file pone.0040481.s003.doc]

Table S3. Primers used for RT-PCR analyses

| Gene symbol | Reference sequence | Forward primer sequence (5’-3’) | Reverse primer sequence (5’-3’) | Amplicon size (bp) | Amplification  cycles |
| --- | --- | --- | --- | --- | --- |
| *Acer1* | [NM_175731](javascript:if(window.name=='') { window.location.href='./nil'; } else { dynPopitupType('NCBI_REFSEQ__TRANSCRIPT', 'http://www.ncbi.nlm.nih.gov/entrez/query.fcgi?db=nucleotide&term=NM_175731'); }) | TCTCCATCCTGTGGTTGTTG | TTATGAGGACGTGCCAAATG | 372 | 36 |
| *Agtr1* | [NM_177322](javascript:if(window.name=='') { window.location.href='./nil'; } else { dynPopitupType('NCBI_REFSEQ__TRANSCRIPT', 'http://www.ncbi.nlm.nih.gov/entrez/query.fcgi?db=nucleotide&term=NM_177322'); }) | GGAAACAGCTTGGTGGTGAT | TGGTTAGGCCCAGTCCTATG | 463 | 36 |
| *Amy2a5* | [NM_009669](javascript:if(window.name=='') { window.location.href='./nil'; } else { dynPopitupType('NCBI_REFSEQ__TRANSCRIPT', 'http://www.ncbi.nlm.nih.gov/entrez/query.fcgi?db=nucleotide&term=NM_009669'); }) | TTCTGCTGCTTTCCCTCATT | CATTGTTGCACCTTGTCACC | 300 | 40 |
| *Atp5g2* | [NM_026468](javascript:if(window.name=='') { window.location.href='./nil'; } else { dynPopitupType('NCBI_REFSEQ__TRANSCRIPT', 'http://www.ncbi.nlm.nih.gov/entrez/query.fcgi?db=nucleotide&term=NM_026468'); }) | GATGAGAGCCTCAGCAGCTT | CCAGAATCGCGTAGGAGAAG | 244 | 36 |
| *Atrx* | [NM_009530](javascript:if(window.name=='') { window.location.href='./nil'; } else { dynPopitupType('NCBI_REFSEQ__TRANSCRIPT', 'http://www.ncbi.nlm.nih.gov/entrez/query.fcgi?db=nucleotide&term=NM_009530'); }) | AAGAAGTCCCCAGGTTCAGG | AGGGCCTGGGTCAACTAAAG | 393 | 36 |
| *β-actin* | [NM_007393](javascript:if(window.name=='') { window.location.href='./nil'; } else { dynPopitupType('NCBI_REFSEQ__TRANSCRIPT', 'http://www.ncbi.nlm.nih.gov/entrez/query.fcgi?db=nucleotide&term=NM_007393'); }) | CCTAGGCACCAGGGTGTGAT | TCACGGTTGGCCTTAGGGTT | 239 | 36 |
| *Bc031748* | [NM_146261](javascript:if(window.name=='') { window.location.href='./nil'; } else { dynPopitupType('NCBI_REFSEQ__TRANSCRIPT', 'http://www.ncbi.nlm.nih.gov/entrez/query.fcgi?db=nucleotide&term=NM_146261'); }) | AGGTGAGGGTGCACATAAGG | ATCAACCTGTCCACACGTCA | 425 | 36 |
| *Bex1* | [NM_009052](javascript:if(window.name=='') { window.location.href='./nil'; } else { dynPopitupType('NCBI_REFSEQ__TRANSCRIPT', 'http://www.ncbi.nlm.nih.gov/entrez/query.fcgi?db=nucleotide&term=NM_009052'); }) | AGGAGAAGGCAAGGATAGGC | GCATGAGGCAAAACTCATCA | 411 | 40 |
| *Bmp15* | [NM_009757](javascript:if(window.name=='') { window.location.href='./nil'; } else { dynPopitupType('NCBI_REFSEQ__TRANSCRIPT', 'http://www.ncbi.nlm.nih.gov/entrez/query.fcgi?db=nucleotide&term=NM_009757'); }) | TTTGGATCTGGCCAAAGAAG | GGGAGAAGGCTTTGAGGAAC | 391 | 36 |
| *Brca1* | [NM_009764](javascript:if(window.name=='') { window.location.href='./nil'; } else { dynPopitupType('NCBI_REFSEQ__TRANSCRIPT', 'http://www.ncbi.nlm.nih.gov/entrez/query.fcgi?db=nucleotide&term=NM_009764'); }) | GAACAGGATCCGCTCAGTGT | CTTCTTGACAGGGCACAGGT | 402 | 36 |
| *Bub1b* | [NM_009773](javascript:if(window.name=='') { window.location.href='./nil'; } else { dynPopitupType('NCBI_REFSEQ__TRANSCRIPT', 'http://www.ncbi.nlm.nih.gov/entrez/query.fcgi?db=nucleotide&term=NM_009773'); }) | AAGAGGAGGAGGCTTTGGAG | CCTGGCTGAAGTTCGTTCTC | 298 | 36 |
| *Ddx3y* | [NM_012008](javascript:if(window.name=='') { window.location.href='./nil'; } else { dynPopitupType('NCBI_REFSEQ__TRANSCRIPT', 'http://www.ncbi.nlm.nih.gov/entrez/query.fcgi?db=nucleotide&term=NM_012008'); }) | GCAAATTTGAACGGAGTGGT | TTACGGCGACCATATCTTCC | 442 | 36 |
| *Dub2* | [NM_010089](javascript:if(window.name=='') { window.location.href='./nil'; } else { dynPopitupType('NCBI_REFSEQ__TRANSCRIPT', 'http://www.ncbi.nlm.nih.gov/entrez/query.fcgi?db=nucleotide&term=NM_010089'); }) | CTGTCCTGGTCCATGAAGGT | CCCAAGTTTCTGGTGGTTCT | 446 | 36 |
| *Eif2s3x* | [NM_012010](javascript:if(window.name=='') { window.location.href='./nil'; } else { dynPopitupType('NCBI_REFSEQ__TRANSCRIPT', 'http://www.ncbi.nlm.nih.gov/entrez/query.fcgi?db=nucleotide&term=NM_012010'); }) | TCTCGCCACATTGGATGTTA | AGGGCATGATTCGTTACCAG | 442 | 36 |
| *Eif2s3y* | [NM_012011](javascript:if(window.name=='') { window.location.href='./nil'; } else { dynPopitupType('NCBI_REFSEQ__TRANSCRIPT', 'http://www.ncbi.nlm.nih.gov/entrez/query.fcgi?db=nucleotide&term=NM_012011'); }) | TCAGTATGCTGCTCCAGGTG | TCGTCGGCTTAGAGCAATTT | 355 | 36 |
| *Epas1* | [NM_010137](javascript:if(window.name=='') { window.location.href='./nil'; } else { dynPopitupType('NCBI_REFSEQ__TRANSCRIPT', 'http://www.ncbi.nlm.nih.gov/entrez/query.fcgi?db=nucleotide&term=NM_010137'); }) | CCGCCACAGATCTCTCTTTC | AGACCTGCTACCCTCCACCT | 426 | 36 |
| *Ggta1* | [NM_010283](javascript:if(window.name=='') { window.location.href='./nil'; } else { dynPopitupType('NCBI_REFSEQ__TRANSCRIPT', 'http://www.ncbi.nlm.nih.gov/entrez/query.fcgi?db=nucleotide&term=NM_010283'); }) | GCTGCTGAAGGTTCTGTTCC | AAACCCCTCATGCTGTTCAC | 449 | 36 |
| *Gja1* | [NM_010288](javascript:if(window.name=='') { window.location.href='./nil'; } else { dynPopitupType('NCBI_REFSEQ__TRANSCRIPT', 'http://www.ncbi.nlm.nih.gov/entrez/query.fcgi?db=nucleotide&term=NM_010288'); }) | AACAGTCTGCCTTTCGCTGT | TGAGAGGAAGCAGTCCACCT | 461 | 36 |
| *Hprt* | [NM_013556](javascript:if(window.name=='') { window.location.href='./nil'; } else { dynPopitupType('NCBI_REFSEQ__TRANSCRIPT', 'http://www.ncbi.nlm.nih.gov/entrez/query.fcgi?db=nucleotide&term=NM_013556'); }) | CCTGCTGGATTACATTAAAG  CACTG | GTCAAGGGCATATCCAACAAC  AAAC | 352 | 36 |
| *Itm2a* | [NM_008409](javascript:if(window.name=='') { window.location.href='./nil'; } else { dynPopitupType('NCBI_REFSEQ__TRANSCRIPT', 'http://www.ncbi.nlm.nih.gov/entrez/query.fcgi?db=nucleotide&term=NM_008409'); }) | CGTGAGGATGACAACATTGC | ACCAGGTCTTCACGAACCAC | 248 | 36 |
| Gene symbol | Reference sequence | Forward primer sequence (5’-3’) | Reverse primer sequence (5’-3’) | Amplicon size (bp) | Amplification  cycles |
| *Pgk1* | [NM_008828](javascript:if(window.name=='') { window.location.href='./nil'; } else { dynPopitupType('NCBI_REFSEQ__TRANSCRIPT', 'http://www.ncbi.nlm.nih.gov/entrez/query.fcgi?db=nucleotide&term=NM_008828'); }) | TGGACAAGCTGGACGTGAA | GAGGCTCGGAAAGCATCAAT | 433 | 36 |
| *Rbmy1* | [NM_001166384](javascript:if(window.name=='') { window.location.href='./nil'; } else { dynPopitupType('NCBI_REFSEQ__TRANSCRIPT', 'http://www.ncbi.nlm.nih.gov/entrez/query.fcgi?db=nucleotide&term=NM_001166384'); }) | AATATGCCAAGAGGAGAGCC | GATGGTGCCTCATGGAATCT | 275 | 36 |
| *Sry* | [NM_011564](javascript:if(window.name=='') { window.location.href='./nil'; } else { dynPopitupType('NCBI_REFSEQ__TRANSCRIPT', 'http://www.ncbi.nlm.nih.gov/entrez/query.fcgi?db=nucleotide&term=NM_011564'); }) | CTGGTGACAATTGTCTAGAG | TGTGGGTTCCTGTCCCACTG | 349 | 30 |
| *Ssty1* | [NM_009220](javascript:if(window.name=='') { window.location.href='./nil'; } else { dynPopitupType('NCBI_REFSEQ__TRANSCRIPT', 'http://www.ncbi.nlm.nih.gov/entrez/query.fcgi?db=nucleotide&term=NM_009220'); }) | TCCAGCTCTCTATGCTTATCAGC | CACCCAGTTACCAATCAACACAT | 127 | 30 |
| *Stx19* | [NM_026588](javascript:if(window.name=='') { window.location.href='./nil'; } else { dynPopitupType('NCBI_REFSEQ__TRANSCRIPT', 'http://www.ncbi.nlm.nih.gov/entrez/query.fcgi?db=nucleotide&term=NM_026588'); }) | GGAGCCTGTGGCTTAGACTG | TTCTGTTGCTGTCCAAATCG | 355 | 40 |
| *Ube1x* | [NM_001136085](javascript:if(window.name=='') { window.location.href='./nil'; } else { dynPopitupType('NCBI_REFSEQ__TRANSCRIPT', 'http://www.ncbi.nlm.nih.gov/entrez/query.fcgi?db=nucleotide&term=NM_001136085'); }) | TTGGCTGTGAATTGCTCAAG | GTGGATCCTGGCTAGAGCTG | 438 | 36 |
| *Ube1y1* | [NM_011667](javascript:if(window.name=='') { window.location.href='./nil'; } else { dynPopitupType('NCBI_REFSEQ__TRANSCRIPT', 'http://www.ncbi.nlm.nih.gov/entrez/query.fcgi?db=nucleotide&term=NM_011667'); }) | GACCCCAAGTTCATGGAGC | CCTCCTAGTCCGTATGTCTGAGC | 335 | 36 |
| *Usp9x* | [NM_009481](javascript:if(window.name=='') { window.location.href='./nil'; } else { dynPopitupType('NCBI_REFSEQ__TRANSCRIPT', 'http://www.ncbi.nlm.nih.gov/entrez/query.fcgi?db=nucleotide&term=NM_009481'); }) | AGGAACCTGCATTTCCACAC | GGTCTGGAGAACGAGCAAAG | 453 | 36 |
| *Usp9y* | [NM_148943](javascript:if(window.name=='') { window.location.href='./nil'; } else { dynPopitupType('NCBI_REFSEQ__TRANSCRIPT', 'http://www.ncbi.nlm.nih.gov/entrez/query.fcgi?db=nucleotide&term=NM_148943'); }) | TGAGAGCCAGGGTCAGACTT | ATCCACTCACAGCCTCATCC | 380 | 42 |
| *Uty* | [NM_009484](javascript:if(window.name=='') { window.location.href='./nil'; } else { dynPopitupType('NCBI_REFSEQ__TRANSCRIPT', 'http://www.ncbi.nlm.nih.gov/entrez/query.fcgi?db=nucleotide&term=NM_009484'); }) | AAATGCAGCTCGGACCAAATC | CTGAATGATGTGAAGCTGTC | 272 | 30 |
| *Vasn* | [NM_139307](javascript:if(window.name=='') { window.location.href='./nil'; } else { dynPopitupType('NCBI_REFSEQ__TRANSCRIPT', 'http://www.ncbi.nlm.nih.gov/entrez/query.fcgi?db=nucleotide&term=NM_139307'); }) | AGTACAGGGTTGCCCATCAG | TGGAGATCTCGTGCAGTTTG | 290 | 36 |
| *Wbp5* | [NM_011712](javascript:if(window.name=='') { window.location.href='./nil'; } else { dynPopitupType('NCBI_REFSEQ__TRANSCRIPT', 'http://www.ncbi.nlm.nih.gov/entrez/query.fcgi?db=nucleotide&term=NM_011712'); }) | TACACAACAGGCACCTGAGC | TGGGCAAAGGTCTTATCAGG | 440 | 40 |
| *Xiap* | [NM_009688](javascript:if(window.name=='') { window.location.href='./nil'; } else { dynPopitupType('NCBI_REFSEQ__TRANSCRIPT', 'http://www.ncbi.nlm.nih.gov/entrez/query.fcgi?db=nucleotide&term=NM_009688'); }) | TTGGAACATGGACATCCTCA | CGCCTTAGCTGCTCTTCAGT | 514 | 36 |
| *Xist* | NR_001570 | ACTGCCAGCAGCCTATACAG | GTTGATCCTCGGGTCATTTA | 578 | 36 |
| *Zfy1* | [NM_009570](javascript:if(window.name=='') { window.location.href='./nil'; } else { dynPopitupType('NCBI_REFSEQ__TRANSCRIPT', 'http://www.ncbi.nlm.nih.gov/entrez/query.fcgi?db=nucleotide&term=NM_009570'); }) | GCCAGTGCTCTCTTAAACCAA | TGAGTACACAAAGTCCCAGCA | 386 | 40 |
| *Zfy2* | [NM_009571](javascript:if(window.name=='') { window.location.href='./nil'; } else { dynPopitupType('NCBI_REFSEQ__TRANSCRIPT', 'http://www.ncbi.nlm.nih.gov/entrez/query.fcgi?db=nucleotide&term=NM_009571'); }) | GCCAGTGCTATGTTACACCATG | TCTGTATGCATTGTCCCAGCA | 386 | 40 |
